# Optimising expression of the large dynamic range FRET pair mNeonGreen and superfolder mTurquoise2^ox^ for use in the *Escherichia coli* cytoplasm

**DOI:** 10.1038/s41598-022-22918-2

**Published:** 2022-10-26

**Authors:** Laureen M. Y. Mertens, Tanneke den Blaauwen

**Affiliations:** Bacterial Cell Biology and Physiology, Swammerdam Institute for Life Science, University of Amsterdam, Science Park 904, 1098 XH Amsterdam, The Netherlands

**Keywords:** Expression systems, Bacterial techniques and applications, Cellular microbiology, Translation, Biological fluorescence, Fluorescence imaging, Microbiology techniques

## Abstract

The fluorescent proteins superfolder mTurquoise2^ox^ (sfTq2^ox^) and mNeonGreen function excellently in mammalian cells, but are not well expressed in *E. coli* when forming the N-terminus of constructs. Expression was increased by decreasing structures at the start of their coding sequences in the mRNA. Unfortunately, the expression of mNeonGreen started from methionine at position ten as optimisation introduced an alternative RBS in the GFP N-terminus of mNeonGreen. The original start-codon was not deleted, which caused the expression of isomers starting at the original start-codon and at the start-codon at the beginning of the GFP N-terminus. By omitting the GFP N-terminus of mNeonGreen and optimising the structure of its mRNA, the expression of a mixture of isomers was avoided, and up to ~ 50-fold higher expression rates were achieved for mNeonGreen. Both fluorescent proteins can now be expressed at readily detectable levels in *E. coli* and can be used for various purposes. One explored application is the detection of in-cell protein interactions by FRET. mNeonGreen and sfTq2^ox^ form a FRET pair with a larger dynamic range than commonly used donor–acceptor pairs, allowing for an excellent signal-to-noise ratio, which supports the detection of conformational changes that affect the distance between the interacting proteins.

## Introduction

Fluorescent proteins genetically fused to proteins of interest are widely used in molecular biology to study protein localisation, transport, interactions, dynamics and many other facets of life. Fluorescent proteins are used across all domains of life and in cells of organisms as diverse as bacteria^1^, plants^2^, and humans^3^. Most fluorescent proteins, however, are developed for use in a mammalian context and are in our experience not well expressed in *Escherichia coli* specifically, or bacteria in general. Expanding of available fluorescent proteins (FPs) for use in bacteria allows for more complex experiments, like detecting the concurrent expression of multiple fusions, or for more complex techniques such as FRET, high-resolution microscopy or FRAP -all current methods used by microbiologists working on complex and relevant scientific questions^4–9^.

Our group has previously developed the donor superfolder turquoise 2 ox (sfTq2^ox^), and the acceptor mNeonGreen (mNG) FRET pair for the periplasm^10^. It has a superior dynamic range to previously used FRET pairs for *E. coli*^10^. This FRET pair was also a promising avenue for developing a more sensitive FRET assay for the cytoplasm than the thus far used cytoplasmic donor mKusabira-Orange (mKO) and acceptor mCherry (mCh) FRET pair^11^. The experimentally determined dynamic range of mKO-mCh was 1.5–31%, whereas the dynamic range of sfTq2^ox^-mNG in the cytoplasmic was 1.5–66%^10,11^. A larger dynamic range allows for a better distinction between FRET and no-FRET and, therefore, a better signal-to-noise ratio and more sensitive measurements. The chromophore of the fluorescent proteins is in the centre of their beta-barrel structure. Consequently, the distance between the donor and acceptor chromophore will always be more than approximately 5 nm. The Förster distance R_0_ is the distance between the chromophores that yields 50% FRET. The sfTq2^ox^-mNG pair has a larger R_0_ than the mKO-mCh pair resulting in a better dynamic range.

However, when repurposing the FRET pair sfTq2^ox^ and mNG for the cytoplasm of *E. coli,* we encountered deficient expression of N-terminal cytoplasmic fusions to sfTq2^ox^. So far, in *E. coli*, sfTq2^ox^ was only employed in the periplasmic space or C-terminal fusions. Consequently, the coding sequence for sfTq2^ox^ was never the start of an open reading frame (ORF). It was always preceded by either a signal sequence for its targeting to the Sec-machinery (in the case of N-terminal fusions) or by another protein (C-terminal fusions, including the mNG-sfTq2^ox^ cytoplasmic tandem). sfTq2^ox^ was developed from mTq2^10^, a distant derivate of enhanced GFP (EGFP)^12^, a GFP variant optimised for expression in human cells^3^. As the expression of sfTq2^ox^ for periplasmic purposes was up to par, it was not codon-optimized for *E. coli*. We hypothesise and prove that the initiation of translation is the bottleneck in sfTq2^ox^ expression, and therefore we could enhance expression by optimising specifically the first 20 codons of the protein.

The expression of fluorescent protein fusions is restricted to a small window of appropriate amounts. Too low expression does not result in detectable signals; too high expression of fluorescent protein(fusion)s can be detrimental to cells. There is an additional requirement for a FRET assay: the produced amounts of the two interaction partners should be close to a 1:1 ratio. To fulfil this latter requirement, the expression of mNG had to be increased as well. For our studies of bacterial cell cycle-related events, the fluorescent fusion proteins are expressed from low copy-number plasmids under the control of an IPTG inducible downregulated pTRC99A promoter (P_trcdown_) or by their native promoter (region)^10,11,13^. Using the P_trcdown_ expression system, we optimised the expression of the sfTq2^ox^-mNG FRET pair. During the optimisation of the expression of these proteins, we confirmed that GFP-based terminal fragments added to many fluorescent proteins might provide alternative ribosome binding sites (RBSs) and lead to the expression of isoforms. A Standard Operating Procedure (SOP) for optimising fluorescent protein expression in bacteria has been formulated, (Supplementary Fig. S1).

The broader purpose of this manuscript is to create awareness of a relatively easy solution to common problems amongst scientists working with fluorescent proteins in bacteria and possibly archaea. Observing that the expression of a fluorescent protein does not result in detectable fluorescence is now often a dead end for scientists. A problem which we demonstrate in this work can be easily overcome, resulting in dramatic increases in expression levels.

## Results

### Increasing sfTq2^ox^ for N-terminal, cytoplasmic expression

**Figure 1:**
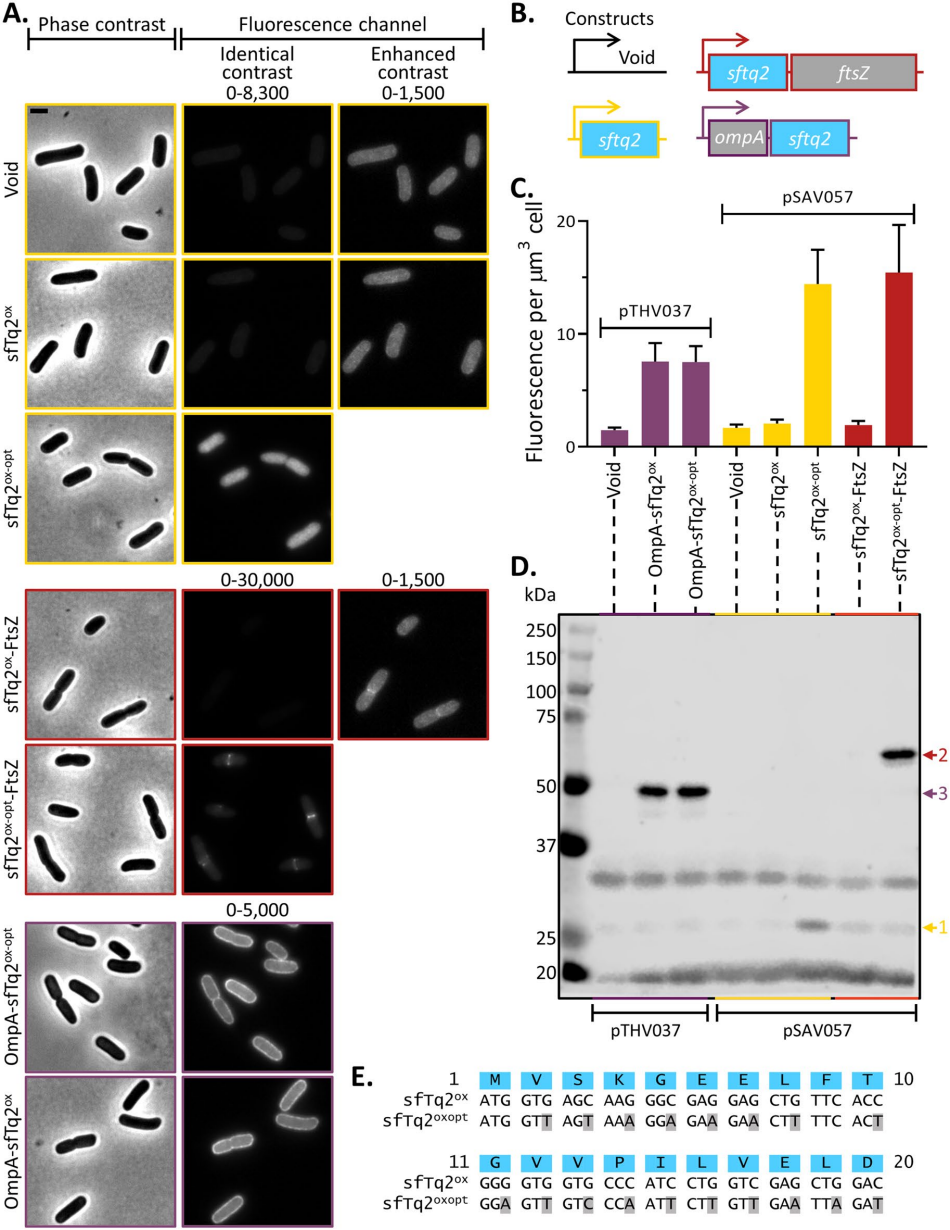
N-terminally optimised sfTq2^oxopt^ is expressed better than sfTq2^ox^. (**A**) Microscopy images, Phase-contrast images are shown on the left, with the corresponding fluorescence images on the right. The numbers above the fluorescence images are the brightness and contrast settings for the image. Scale bar equals 2 µm; (**B**) Graphic representation of the constructs tested in this panel; (**C**) Fluorescence per µm^3^ cell, based on microscopy of 650–770 cells, the bar represents mean value and error bars standard deviation; (**D**) Western blot detecting sfTq2^ox(opt)^ with αGFP reveals higher expression levels of unfused sfTq2^oxopt^ and sfTq2^oxopt^-FtsZ compared to unfused and FtsZ-fused sfTq2^ox^. On the contrary, the fusion to the C-terminus of OmpA1-177 did not cause increased expression. Original blots shown in Supplementary Fig. S2; E. Alignment of sequences of sfTq2^ox^ and sfTq2^oxopt^, mutations are based on *gfp-mut2*^40^, shown with grey highlighting.

The low expression level of open reading frames (ORFs) starting with sfTq2^ox^ was first observed for the fusion sfTq2^ox^-FtsZ (Fig. 1A, B). Several successful N-terminal fusions to the bacterial cell division protein FtsZ that forms a ring structure at midcell have been made before to other fluorescent proteins such as mCherry^11^, SYFP2^11^ and mNG (unpublished). However, sfTq2^ox^-FtsZ fusions did not result in apparent midcell localisation, nor did the detected fluorescence level increase above the autofluorescence of cells transformed with a void plasmid (Fig. 1A, C). This was also the case for unfused, cytoplasmic sfTq2^ox^ (Fig. 1A–C). Since expression of C-terminal sfTq2^ox^ fusions in *E. coli* was higher and previous work has shown that synonymous mutations in the N-terminal region of GFP have a serious impact on its expression levels^14^, the codons in the N-terminus of sfTq2^ox^ were exchanged to improve its expression level. sfTq2^ox^ is a far derivative of human-optimized GFP (EGFP), and the amino acids at its N-terminus still match those of EGFP completely^12,15–17^. The first 20 codons of sfTq2^ox^ were replaced by synonymous codons used in *gfp-mut2*, a GFP variant well expressed in *E. coli* (sequences are aligned in Fig. 1E)^1^. The version of sfTq2^ox^ with the first 20 codons replaced by those of *gfp-mut2* is termed sfTq2^oxopt^.

The expression levels of unfused sfTq2^oxopt^ and sfTq2^oxopt^-FtsZ were compared with their sfTq2^ox^ equivalents by quantification of fluorescence microscopy images (Fig. 1A, C) and western blotting (Fig. 1D) of cells grown exponentially in rich medium. The sfTq^oxopt^ variants were expressed much better than the sfTq2^ox^ variants (Fig. 1C). The enhanced brightness image of Fig. 1A shows that the cells expressing sfTq2^ox^ exhibit similar levels of fluorescence as the cells with the void plasmid and that sfTq2^ox^-FtsZ expressing cells show some midcell localisation, albeit scarcely distinguishable from the cell’s autofluorescence. The increase in expression of constructs commencing with sfTq2^oxopt^ is sufficient to detect fluorescence and to visualise localisation in the cells. Cells still have a regular morphology and grow as wild type, indicating that the expression levels are below a toxic threshold.

The hypothesis that the start of translation was the limiting factor for expressing constructs commencing with sfTq2^ox^, was further examined by introducing sfTq2^oxopt^ in a C-terminal fusion to the outer membrane protein OmpA (Fig. 1B). Since the translation start and amino acids of OmpA-sfTq2^ox^ and OmpA-sfTq2^oxopt^ are the same, their expression level should not be different despite the changes made in the sfTq2^oxopt^. Indeed, the images in the lowest panel of Fig. 1A and their quantification in Fig. 1C show that the amount of fluorescence signal for those fusions remained similar. This is further confirmed by the bands with similar intensity in the western blot in Fig. 1D.

The mutations introduced in sfTq2^ox^ to create sfTq2^oxopt^ all but one increased the amount of A/T-nucleotides and reduced the amount of G/C-nucleotides. This likely lowers secondary structures in this part of the mRNA and facilitates the binding of ribosomes and translation initiation. To verify whether the mutations impact the secondary structure, the 5′ UTR sequence until the fiftieth codon were entered in the RBS Calculator tool provided by SalisLab.net^18^. Results (Supplementary Table S1) show that secondary structures surrounding the start codon in the unbound mRNA (ΔG_mRNA_) are less stable for sfTq2^oxopt^ than for sfTq2^ox^. Also, the transition from unbound mRNA to ribosome-bound mRNA (ΔG_total_) requires less energy input for sfTq2^oxopt^ than sfTq2^ox^.

In conclusion, the expression of N-terminal sfTq2^ox^ fusion has been improved to a detectable level by introducing alternative codons derived from *gfp-mut2*. This increase in expression occurs only when sfTq2^oxopt^ forms the N-terminus of the expression fusion, not when it is present as a C-terminal fusion. This result agrees with the strong correlation of a lower GC-content near the start codon with increased expression of GFP^14^. General recommendations for optimising the expression of EGFP derived fluorescent proteins in bacteria can be found in Supplementary Fig. S1.

### Optimising the N-terminus of mNeonGreen for better expression

mNG was developed from lanYFP, found in *Branchiostoma lanceolatum*^19^. During the development of mNG, short termini of EGFP were added to the termini of lanYFP (Fig. 1A, C). This is a common intervention, also found in (amongst others) mCh, its derivatives and other members of the mFruits family of fluorescent proteins^20^. For the comprehensibility of the present study, the different versions of mNG discussed are named mNG-version1-6 (abbreviated as mNG-v1-6).

As with sfTq2^ox^, C-terminal fusions of mNG (mNG-v1) were well expressed^10,13^. Constructs with mNG (the FRET partner of sfTq2^ox^) at the N-terminus were initially expressed at undetectable levels in *E. coli*. This was overcome by omitting the GFP-derived N-terminus of mNG, resulting in mNG-v2^13^. mNG-v2 was also used in the cytoplasmic tandem to prove that the dynamic range of mNG-sfTq2^ox^ was 1.5–66%^10,11^. However, to match the expression level of constructs commencing with sfTq2^oxopt^, a requirement for FRET measurements, this intervention was not sufficient. Therefore, the expression of mNG had to be further enhanced.

**Figure 2:**
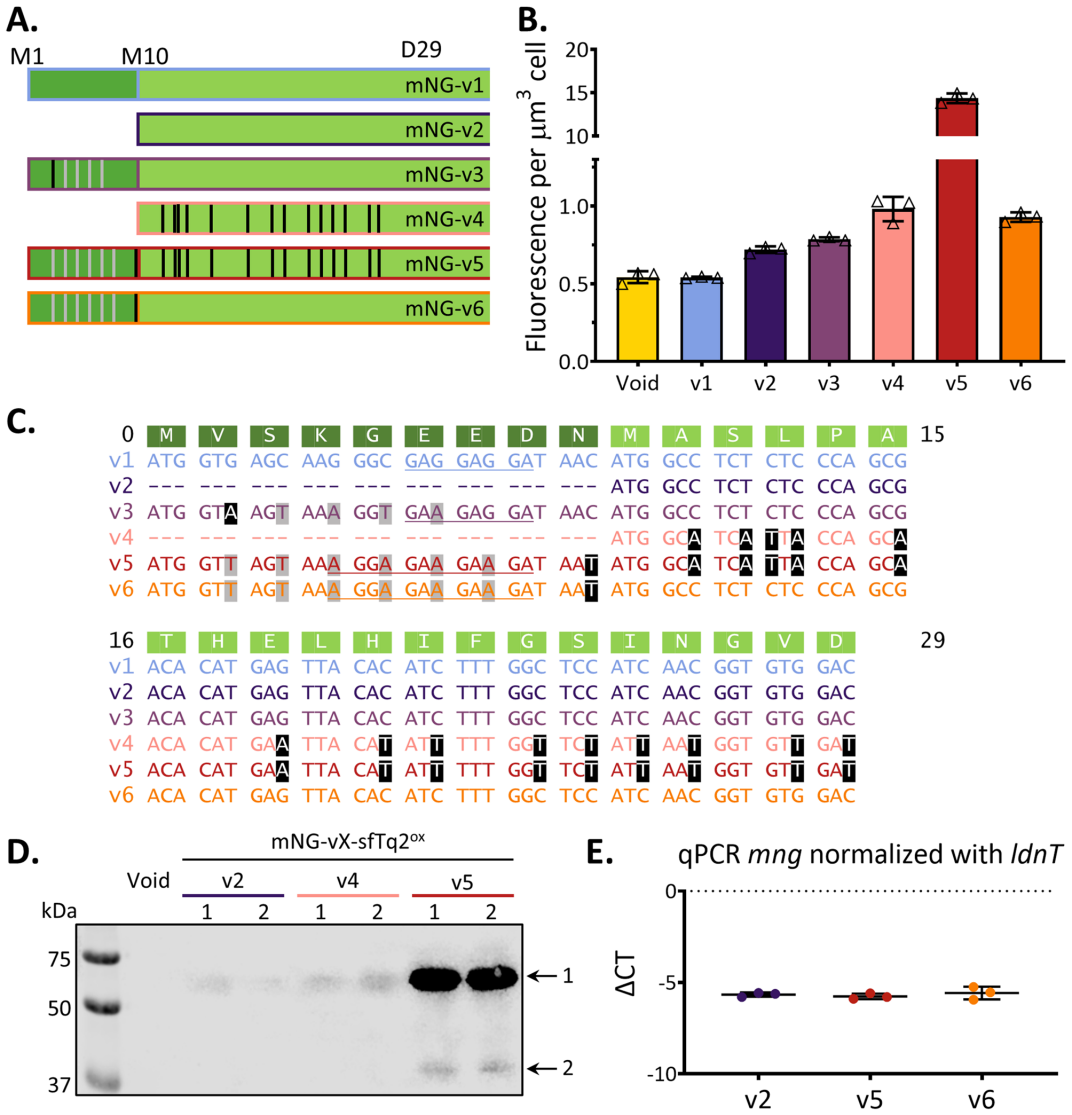
Mutations to the N-terminus of mNeonGreen impact expression levels. (**A**) Graphic representation of the different N-termini of mNG that were tested. Dark green amino acid blocks represent the GFP-derived N-terminus, bright green is the sequence-specific to mNG, grey vertical lines indicate silent mutations derived from *gfp-mut2*, and black vertical lines represent silent mutations exchanging G or C-nucleotides by A or T nucleotides; (**B**) Fluorescence per µm^3^ cell for different mNG variants, based on microscopy of three separate LMC500 clones per construct, 325–633 cells per construct were analysed. The bar represents mean value and error bars standard deviation. Representative microscopy images can be found in Supplementary Fig. S3; (**C**) Sequence of different N-termini of mNG, grey nucleotides represent *gfp-mut2* derived silent mutations, black nucleotides silent mutations exchanging G or C-nucleotides by A or T nucleotides; (**D**) Western blot confirming increase expression of constructs starting with mNG-v5, with anti-mNG. Arrow 1 indicates the height of the mNG-sfTq2^ox^ protein; arrow 2 indicates a putative degradation product. Original western blots can be found in Supplementary Fig. S2; (**E**) Normalized threshold cycle qPCR on three mNG variants.

mNG-v1 contains the commonly used N-terminal (E)GFP terminus MVSKGEEDN. The mNG-v1 expression has also been increased by exchanging codons for V2 to E6 with synonymous codons with less C and G nucleotides, resulting in mNG-v3 (Fig. 2A, C show sequences)^21^. mNG-v3 was developed for use in promotor-reporter assays. mNG-v2 was previously used for localisation studies and as the N-terminus of an mNG-sfTq2^ox^ tandem for FRET. mNG-v3 and mNG-v2 were expressed at similar levels (Fig. 2B and Supplementary Fig. S3). mNG-v2 was used as the starting point for optimisation, as it has already performed well in N-terminal fusions for localisation and tandem-FRET purposes. Codons A11 to D20 in mNG-v2 were optimised by replacing C and G nucleotides with A or T nucleotides in synonymous codons. The resulting mNG-v4 was expressed at a higher level than mNG-v2 and mNG-v3. The signal of a mid-cell localising fusion was also better (Supplementary Fig. S4). However, when used as a FRET pair with sfTq2^oxopt^, the expression of mNG-v4 was not sufficiently improved to match the expression level of sfTq2^oxopt^.

Therefore, we re-introduced the N-terminal GFP sequence to mNG-v4, but now encoded by the codons used in *gfp-mut2* and AT-optimised one of the two spacer codons (N9, see Fig. 2A, C). The resulting mNG-v5 showed a much higher fluorescence per µm^3^ cell (Fig. 2B and Supplementary Fig. S3) than mNG-v4. mNG-v5 was then used as a FRET acceptor for sfTq2^oxopt^. The expression of mNG-v5 was now too high for FRET with sfTq2^oxopt^. To reduce the expression of mNG-v5 and obtain a ratio of approximately 1:1 with sfTq^oxopt^, the optimised GFP N-terminus was combined with non-optimised codons A11-D29, resulting in mNG-v6 (Fig. 2A, C). This optimisation resulted in a reduced expression level similar to that of mNG-v4 (Fig. 2B and Supplementary Fig. S3), and mNG-v6 is therefore unsuitable for FRET with sfTq2^oxopt^.

Before further steps were taken to optimise mNG expression levels suitable for FRET with sfTq2^oxopt^, it was established that the differences in expression were not caused by differences in mRNA concentration. A western blot confirmed that the increase in fluorescence detected for mNG-v5 was due to more proteins (Fig. 2D). Additionally, a qPCR was performed on whole RNA-extract from cultures expressing mNG-v2, mNG-v5 and mNG-v6. Differences among the normalized threshold cycles (Fig. 2E) are not significant. The detected threshold sequences for mNG were normalised with established primers^22^ amplifying part of *idnT* (encoding a putative L-idonate and D-gluconate transporter). This confirms that regulation of expression occurs at the level of translation.

When re-analysing the best-expressed mNG so far (mNG-v5), it was noticed that the *gfp-mut2* sequence coding for the GFP peptide contained a putative Shine-Dalgarno (SD) sequence (underlined in Fig. 2A). This was confirmed in silico by the RBS Calculator^18^, predicting an ORF starting at Met10 with a higher predicted translation rate than the ORF commencing at Met1 (Supplementary Table S1 for the predicted translation rates). The original mNG-v1 and mNG-v3 also contain a putative SD sequence upstream of Met10, but their predicted translation rates were far less efficient (Supplementary Table S1). The additional putative SD-sequence in the GFP N-terminus of mNG-v5 could possibly create an alternative, more efficient start of translation.

### Alternative ribosome binding site pivotal to increase in expression of mNeonGreen

**Figure 3:**
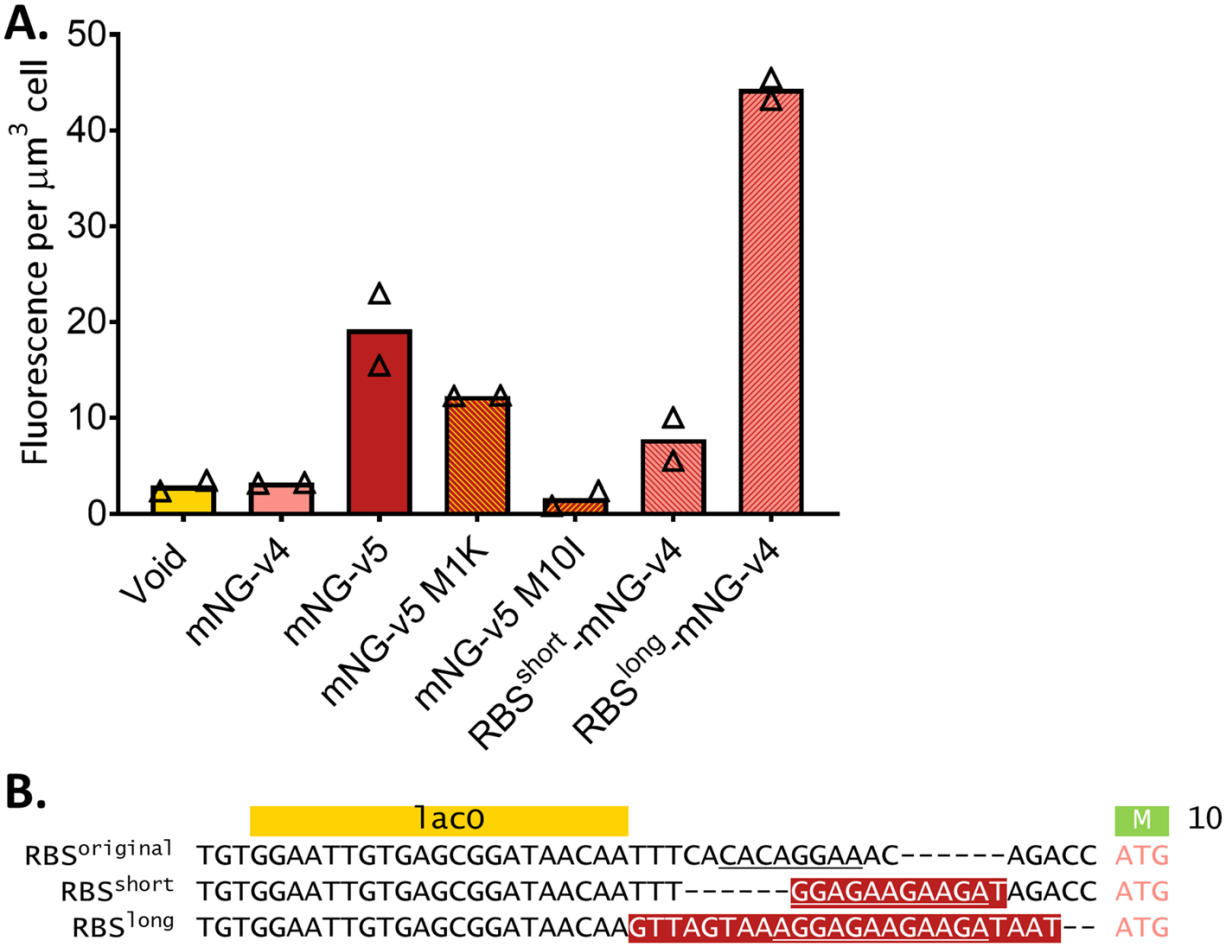
mNG-v5 contains an alternative RBS, and its start of translation is predominantly Met10. (**A**) Fluorescence per µm^3^ cell for different versions of mNG and RBSs of mNG, based on microscopy of two independent experiments, represented by triangles, 185–666 cells per sample were analysed. The bars represent mean value. Representative microscopy images can be found in Supplementary Fig. S1; (**B**) Sequence of the three different 5**′** UTRs to Met10 of three different mNG-v4 constructs. Introduced nucleotides have been highlighted in red, and putative RBSs are underlined.

We speculated that mNG-v5 contains an alternative RBS and that translation started at Met10 rather than at Met1. To prove this, mNG-v5 mutants Met1K (AUG > AAG) and Met10I (AUG > ATA) were constructed, restricting translation to start at either Met10 or Met1, respectively. The fluorescence of strains expressing these mutants was compared with strains expressing mNG-v5. Figure 3A shows that mNG-v5 M1K is still expressed, albeit not as much as mNG-v5. Cells expressing mNG-v5 M10I did not fluoresce above the background of cells with a void plasmid. mNG-v5 is, to a large extend, expressed from the alternative RBS and the ORF starting at Met10. It confirms the prediction of the RBS Calculator that Met10 is an efficient start of translation (Supplementary Table S1).

Notably, mNG-v6 also contains the alternative RBS but is not expressed as well as mNG-v5 (Fig. 2B, C). This means that in addition to a stronger RBS, AT-optimization of codons A11-D29 is also required to achieve the high expression levels of mNG-v5. We, therefore, continued our search for the desired expression level of mNG by replacing the original RBS of mNG-v4 with a short part (G5-D8) or a long part (V2-N9) of mNG-v5. Creating RBS^short^-mNG-v4 and RBS^long^-mNG-v4 (sequences in Fig. 3B). RBS^short^ misses part of the putative RBS sequences and could therefore regulate mNG expression to obtain a good ratio with sfTq2^oxopt^. The RBS^short^-mNG-v4 construct resulted indeed in a lowered expression compared to mNG-v5 and is a potential candidate for FRET with sfTq2^oxopt^. RBS^long^-mNG-v4 resulted in more than double the measured fluorescence per µm^3^ cell as for mNG-v5 and is therefore not valid for the purpose of a balanced FRET pair. However, it might have a purpose in experiments that require high expression levels, such as reporter fusions. We have drafted a Standard Operating Procedure (Supplementary Fig. S1) for optimising the expression of fluorescent proteins with a GFP-derived N-terminus, like mNG.

### mNG-RBS^short^-v4 and sfTq2^oxopt^ form a balanced FRET pair

**Figure 4:**
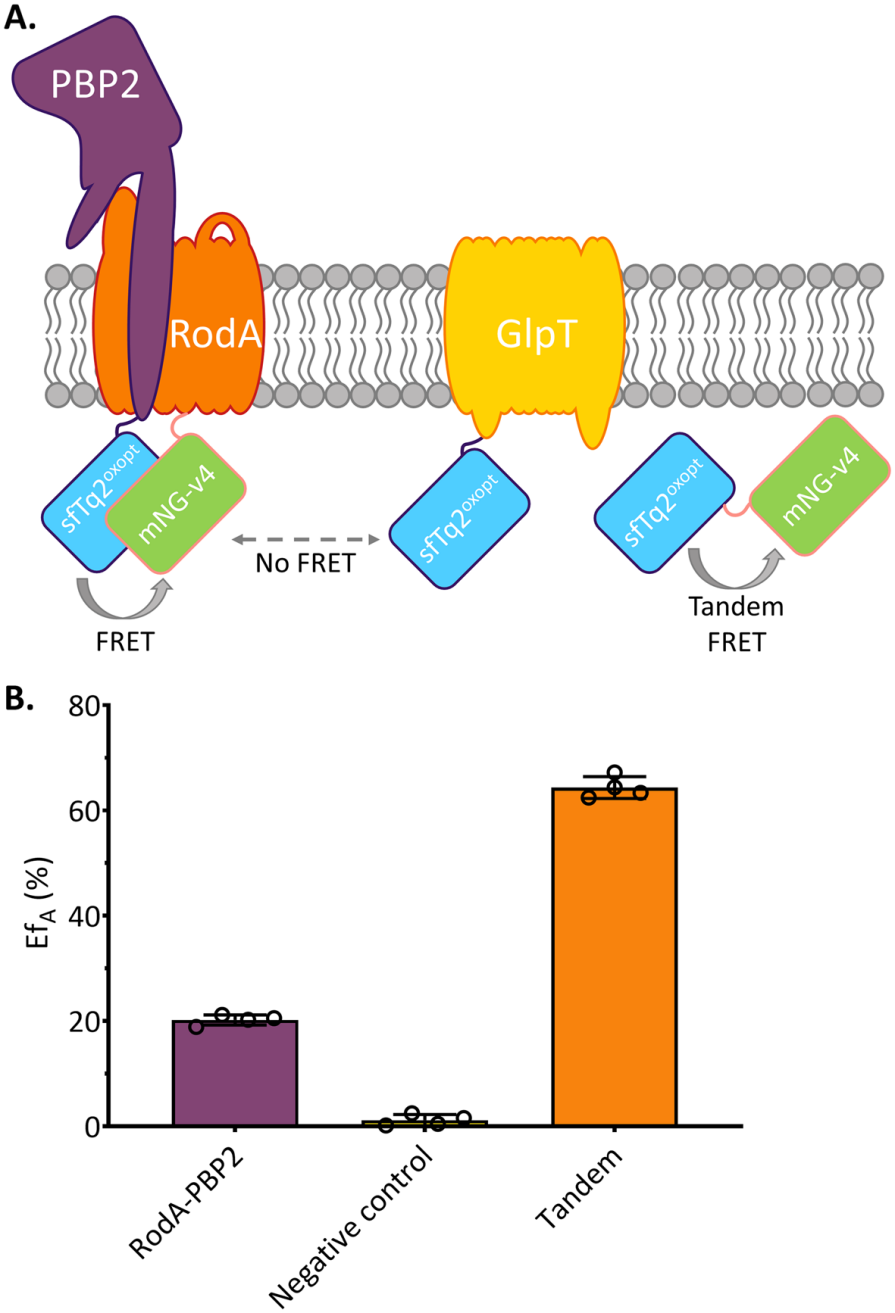
sfTq2^oxopt^ and RBS^short-^mNG-v4 form a balanced FRET pair for the cytoplasm. (**A**) Schematic drawing of the inner membrane with fluorescent fusions to PBP2, RodA and GlpT. The curved arrow represents FRET; dashed arrow indicates that the distance between non-interacting proteins is too large to measure FRET; (**B**) Ef_A_ (%) detected for tandem (RBS^short^-mNG-v4-sfTq2^ox^), negative control (RBSshort-mNG-v4-RodA and sfTq2^oxopt^-GlpT) and biological interaction between RBS^short^-mNG-v4-RodA and sfTq2^oxopt^-PBP2. The bar represents mean value and error bars standard deviation of four replicates. Supplementary Table S3 shows the actual values for Ef_A_. Representative microscopy images can be found in Supplementary Fig. S6 and the unmixing of the individual FRET experiments in Supplementary Fig. S7.

The last step of this project was to confirm that the expression ratio between RBS^short^-mNG-v4 and sfTq2^oxopt^ does allow for FRET measurements. A ~ 1:1 ratio of mNG:sfTq2^ox^ is required to reliably detect FRET, meaning an mNG variant was needed that expressed ~ 3 times fewer proteins than the highly expressed mNG-v5. The previous section showed that RBS^short^-mNG-v4 approximately meets that requirement (Fig. 3A), and it was used in a FRET experiment. PBP2 and RodA have previously been used in successful FRET experiments fused to the mKO-mCh FRET pair yielding an acceptor FRET efficiency (Ef_*A*_, where the increase in fluorescence of the acceptor fluorescent protein is the measure of FRET) of about 13%^23^. The protein fusions sfTq2^oxopt^-PBP2 and RBS^short^-mNG-v4-RodA were constructed to test the new FRET pair. Positive control is the tandem RBSshort-mNG-v4-sfTq2ox and negative control is the non-interacting RBS^short^-mNG-v4-RodA and sfTq2^oxopt^-GlpT were used (schematically shown in Fig. 4A, and Supplementary Table S2). As expected, the Ef_*A*_ for the RodA-PBP2 pair was increased to 20% due to the larger R_0_ of the sfTq2-mNG FRET pair compared to the mKO-mCh FRET pair (Fig. 4B and Supplementary Table S3). The FRET efficiency based on the donor (using the decrease in donor fluorescent protein fluorescence as a measure for FRET) was 25%: indicating that the sfTq2 to mNG ratio was 0.8, which was sufficiently close to the desired 1:1 ratio to function as FRET pair to study protein interactions.

In conclusion, a more efficiently expressed variant sfTq2^ox^ and a matching mNG have been constructed that can function to study the interaction between proteins in the cytoplasm of *E. coli* and possibly other bacterial species. In addition, an exceptionally well expressed mNG version was constructed (RBS^long^-mNG-v4) that could be useful as promoter reporters or whenever higher expression rates are required.

## Discussion

Fluorescent proteins have been applied across all domains of life. This is possible since the genetic code, converted from DNA to RNA, to proteins, as described by the central dogma^24^ of molecular biology, is virtually universal to all known life forms. However, the types of machinery and, therefore, (regulatory) mechanisms of protein expression vary amongst the different domains of life. In eukaryotes -with some exceptions like internal ribosome entry sites (IRES^25^)- translation is initiated by the binding of a ribosome to the CAP at the 5′ terminus of an mRNA and its subsequent scanning for the first start-codon, where translation starts^26^. In bacteria, the ribosomal subunits assemble to form a functional ribosome by the complementarity of the anti-SD sequence on the rRNA to an SD sequence on the mRNA upstream of an ORF^26^. These SD sequences can be located near the 5′ end of an mRNA but can be found along the entire length of mRNAs (allowing for the polycistronic nature of bacterial mRNAs). These differences have now been proven to be problematic in the heterologous expression of fluorescent proteins in bacteria.

Increasing the expression of the EGFP-derived sfTq2^ox^ in *E. coli* was relatively easy. Rather than replacing all codons in such a protein, this study confirms^14^ that replacing only the first twenty codons with synonymous codons from *gfp-mut2* is sufficient to increase the expression of translational units starting with such fluorescent proteins. The codon replacement did not result in the most frequently used codons but decreased the use of C- and G-nucleotides and increased A- and T-nucleotides usage. As reported by Kudla and colleagues, such replacements in the vicinity of the start-codon correlate with an increase in translation initiation, likely through a decrease in strong mRNA structures near the start-codon^14^. The sequence of the first 20 codons in *gfp-mut2* is a generally applicable sequence which can be used to increase the expression of GFP-derived fluorescent proteins in *E. coli*. Such mutations can easily be introduced with mutagenesis primers using various PCR techniques.

As most fluorescent proteins are initially developed for applications in eukaryotic species (particularly humans and mice), researchers often overlook bacterial-specific requirements or intricacies. The introduction of synonymous codons with an increase in AT-content in the GFP N-terminus added to many non-GFP-derived fluorescent proteins^19,20,27,28^, like mNG, may result in the expression of unwanted isoforms. The introduced GFP N-terminus is MVSKGEEXN (where the X is either L, D or N) and the added GFP C-terminus GMDLYK. Specifically, the KGEEX coding nucleotides provide a putative RBS. The rudimental methionine at position ten, present in mNG^19^, RFP1.3-derivates including mCherry^20^, mFruits^20,27^ and many others^28^, will not be an alternative start of translation in eukaryotes, but it can be in bacteria. Ribosome assembly in bacteria is not restricted to the 5′ end of mRNAs. These N-termini, therefore, introduce an additional RBS at an opportune distance from a rudimental methionine^29^. This can lead to the expression of 9 amino acids shorter isoforms or even the translation of non-fused fluorescent proteins in C-terminal fusions.

Coinciding with our observation of the expression of mNG from a rudimental methionine at the end of a GFP-derived N-terminus, it was also described for mCherry in a preprint by Fages-Lartaud and colleagues^30^. These scientists used an *E. coli* optimised version of mCh, which contained a similar RBS upstream of Met10. Also, coinciding with the preparations of this manuscript, researchers optimised mNG expression for *Saccharomyces cerevisiae* by replacing codons to match the most used codons in *S. cerevisiae* (ymNG_WT_) and subsequently by decreasing structures in the mRNA coding for the mNG N-terminus (ymNG_RM_)^31^. They also introduced ymNG_RM_ in *E. coli* and observed an increase in expression. However, the codon replacements made to decrease structures in the 5′ end of the mRNA are likely introducing an alternative RBS so translation can start from Met10, rather than an increased expression of full-length mNG. We verified this in silico using the RBS predictor^18^ and found that, indeed Met10 is a more likely start of translation than Met1 (Supplementary Table S1).

The expression of a 9-amino acid shorter isoform is not problematic by itself: proteins are still fluorescent, and the GFP N-terminus is not likely to have any effects on other behaviours of the protein. However, there are two arguments why one would want to avoid this. The first being that expressing a mixture of two isoforms—and not knowing which ratio is inept and can be easily avoided by omitting the GFP N-terminus altogether. The second being that in C-terminal fusions, one can theoretically express a relatively large amount of unfused fluorescent protein (starting at Met10 of the fluorescent protein) and not only the expression of the intended fusions protein. This leads to a large amount of background fluorescence. This second problem was not addressed in this study, but researchers should be aware of this risk.

To avoid the expression of isoforms and uncertainty over the exact start of translation, we propose to abolish and/or adapt these GFP-derived peptide linkers at the N-termini of fluorescent proteins when working in bacteria. In N-terminal fusions (FP-protein), the GFP-derived sequence has no function and could be abolished without any consequences.

Another concern we wish to address is that it is often (almost) undecipherable from publications whether fluorescent proteins have been codon-optimized and to what extent. The amino acid sequence and, therefore the resulting protein may be the same, but as this study and the work of Fages-Lartaud and colleagues^30^ shows: it does matter and should be reported more clearly. sfTq2^ox^ and sfTq2^oxopt^ have the same amino acid sequence and can therefore consider to be the same protein: however, their expression in *E. coli* differs hugely. In the case of mNG-v5 versus mNG-v1, the introduction of synonymous codons leads to the expression of two different proteins, emphasising that the current naming system for proteins not allowing for differentiating between those two is too limited. In addition, the recent finding that synonymous mutations often impact fitness as much as non-synonymous mutations in yeast implicates that codon usage has a more significant impact than has been considered so far^32^.

A complicating factor in obtaining a good ratio between mNG and sfTq2^ox^ for FRET is the different effects of fixation on sfTq2^oxopt^ and mNG. It was previously shown that sfTq2^ox^ continues maturing to its fluorescent state after fixation^10^. Cells expressing mNG, however lose approximately 60% of their fluorescence per µm^3^ cell upon fixation and only reach about 70% of the fluorescence per µm^3^ cell volume after overnight maturation (Supplementary Fig. S8). This means that 30% of mNG molecules do not recover to a fluorescent state after fixation. A 1:1 ratio of the fluorescent proteins, in this case, does therefore not mean that there is a 1:1 ratio of proteins that fluoresce. The combination of protein fusions starting with RBS^short^-mNG-v4 and sfTq2^oxopt^, however is sufficiently balanced to be suitable for cytoplasmic FRET.

The improvements in the expression of sfTq2^ox^ and mNG discussed in this manuscript may have broader applications than solely those two fluorescent proteins expressed in specifically *E. coli*. When expression of a fluorescent protein in a bacterium does not result in detectable fluorescence, it may be worth attempting to increase its expression. In Supplementary Fig. S1, the reader can find a flowchart describing steps to take, resources and some potential pitfalls when optimizing expression of fluorescent protein expression for bacterial species, based on this work. The SOP is centred around what worked for *E. coli* but it is constructed in such a manner that it can also be used as a starting point for improving the expression of FPs in other bacterial species. Since translation by the kingdoms of bacteria and archaea is extremely diverse^33^, we cannot guarantee its general applicability. Even in the well-studied bacterium *E. coli,* precise predictions about translation rates are difficult—as evidenced by the deviations between the predicted translation rates (Supplementary Table S1) and the measured fluorescence (Figs. 1, 2, 3). Amongst different bacterial species, more factors have to be taken into account (e.g. some species express mainly leaderless mRNAs, some almost none)^33^. The anti-SD sequence is quite strongly conserved, whereas enormous diversity amongst bacterial species SDs exists, which implies that our findings for the alternative RBS found in the first nine codons of mNG (and other non-GFP derived FPs) is relevant for some, but not all bacterial species.

## Methods

### Strains and growth conditions

This study used two *E. coli* K-12 strains (described in Table 1). LMC500 was used for the expression of FP(-fusion)s, for both establishing expression levels and the FRET assay. For the generation and selection of cloning products, DH5α was utilised.

**Table 1:** Strains used in this study.

Strain	Genotype	
LMC500	MC4100*, F-, araD139, Δ(argF-lac)U169, deoC1, flbB5301, lysA1, ptsF25, rbsR, relA1, rpsL150*	^41^
DH5α	*F-, supE44 ΔlacU169 (Ф80lacZΔM15) hsdR17 recA1 endA1 gyrA96, thi-1 relA1*	Bethesda Research Laboratories (1986)

Strains were grown in either minimal GB1 medium (1 L contains: 4 g glucose (Roth, Karlsruhe, Germany), 4.83 g K_2_HPO_4_ (VWR), 2.95 g KH_2_PO_4_ (Fisher Chemical), 1.05 g (NH_4_)_2_SO_4_ (Sigma-Aldrich), 0.10 g MgSO_4_·7H_2_O (Roth), 0.28 mg FeSO_4_·7H_2_O (Sigma-Aldrich), 7.1 mg Ca(NO_3_)_2_·4H_2_O (Sigma-Aldrich), 4 mg thiamine (Sigma) and 50 mg lysine (Sigma)) at 28 °C for steady-state growth required for FRET experiments, or in rich medium (TY, 1 L contains: 10 g tryptone (Duchefa), 5 g yeast extract (Fisher Bioreagents) and 5 g NaCl (Acros Organics)) at 37 °C for all other purposes. Liquid cultures were grown under constant agitation (200 rpm), whereas media were solidified for selective growth after transformations by adding 20 g agar (Sphaero Q) per litre medium. When needed for plasmid maintenance, media were supplemented with relevant antibiotics: 100 µg/L ampicillin (Roth) and 25 µg/L chloramphenicol (Sigma). The expression of proteins from plasmids was induced with 15 or 50 µM isopropyl ß-D-1-thiogalactopyranoside (IPTG, Duchefa).

### Plasmids and plasmid construction

All plasmids and primers can be found in Supplementary Tables S4 and S5, respectively.

Site-directed mutagenesis by inverse PCR was performed on plasmids to introduce mutations in sfTq2^ox^, and mNG and replace RBSs^34^. To overcome primer-dimerization issues, mutagenesis PCR was performed in two stages. The nine first PCR cycles were performed at low annealing temperature, allowing the mutagenesis primers to anneal to their mismatched template. These were followed by 26 cycles where the annealing temperature was increased for primer binding to the fully matching templates generated in the first nine cycles. The PCR product was isolated and digested with DpnI (New England Biolabs) to degrade the template plasmid. Circularisation was realized upon transformation in DH5α cells as reported before^34^.

Other plasmids were constructed by restriction-ligation cloning (Supplementary Table S4). When necessary for efficiency or introduction of alternative restriction sites, inserts were first amplified by PCR prior to digestion with restriction enzymes (all from New England Biolabs). Ligation was performed with T4 DNA ligase (New England Biolabs), and the product was selected by the transformation in DH5α cells.

Plasmid constructs were also generated with Gibson assembly when appropriate (Supplementary Table S4). PCR reactions generating fragments with matching overhangs were followed by DpnI digestion and band excision from agarose gels. Assembly was performed in homemade Gibson mix (all components from NEB), and successful products were subsequently selected by the transformation in DH5α.

All cloning products were checked by sequencing with the mix2seq kit (Eurofins) before subsequent use in experiments.

### Comparative expression experiments

Experiments comparing FP expression levels were all performed in a rich medium. Overnight cultures were diluted 1:500 in 25 ml of fresh medium, grown to mid-exponential phase (OD_600_ = 0.2–0.3), diluted to an OD_600_ of 0.04 and subsequently induced with 15 µM IPTG. After two mass doublings, living cells were harvested for microscopy and western blot analysis. The remainder of the culture was fixed by adding 2.8% formaldehyde and 0.04% glutardialdehyde (final concentrations).

Living cells intended for microscopy were washed twice in Milli-Q water to remove the yellow background signal stemming from the TY medium. Living cells for western blot analysis were pelleted by centrifugation and frozen at -20 °C after the supernatant was removed. After adding fixatives to the cultures, cultures were fixed for 30 min, under constant agitation. Cultures were subsequently washed in PBS (pH = 7.2), a fraction of each sample was imaged immediately (termed ‘fixed’), and the remainder was stored in a dark place at room temperature for overnight fluorophore maturation and imaged the next day (termed ‘matured’).

### Microscopy

Microscopy slides were prepared by trapping cell suspensions between a 1% agarose (Sphaero Q) slab on an object glass and a cover slip^35^. For living cells, this agarose was added to Milli-Q water, and for fixed cells to PBS, pH = 7.2. Cells were imaged in a phase contrast and fluorescence microscopy channel with an Olympus BX-60 microscope (Tokyo, Japan), equipped with a Hamamatsu ORCAFlash-4.0LT CMOS camera (Hamamatsu, Naka-ku, Japan) and a UPlanApo 100×/N.A. 1.35 oil Iris Ph3 objective. The following filter cubes were used to imaging sfTq2^ox^ expression and mNG expression, respectively: Cyan-GFPv2 (cyan, ex436/20, dic455LP, em480/40) and EN-GFP (green, ex470/40, dic495LP, em525/50).

For two experiments, a different microscope was used. The maturation of mNG-v4 (Supplementary Fig. S8) and the FRET samples (Supplementary Fig. S6) were imaged with a Nikon Eclipse Ti microscope equipped with a C11440-22CU Hamamatsu ORCA camera, a CFI Plan Apochromat DM 100 × oil objective, an Intensilight HG 130 W lamp and the NIS elements software (version 4.20.01). The following filter cubes were used for the imaging of sfTq2^ox^ expression and mNG expression, respectively: CFP MXU96210/C153677 (ex426-446, dic455LP, em460-500) and GFP-B MBE44740 (ex460-500, dic505, em510-560).

Microscopy data were analysed with the ImageJ plugin ‘Coli-Inspector’^36^. The fluorescence per µm^3^ reported as an indication of expression levels of fluorescent proteins is given by ‘Concentration Total’.

### Western blot

Biomass concentrations in frozen cell pellets were equalized by adding appropriate volumes of Lämmli buffer (0.0625 M Tris–HCl (VWR) pH = 6.8, 2% SDS (Merck), 10% glycerol (Biosolve Chimie SARL,), 0.1 M dithiothreitol (Sigma-Aldrich) and 0.001% bromophenol blue (Fisher Scientific)). Samples were kept on ice, heated for 5 min at 99 °C and centrifuged at 20,000 rcf for 5 min before loading. SDS-PAGES were hand-casted (4% stacking gel, 12% separating gel). Proteins were separated by electrophoresis and transferred to a 0.2 µm nitrocellulose membrane (Bio-Rad). sfTq2^ox^ was detected with primary antibody αGFP (1:2000, Fisher Scientific), FtsZ with a polyclonal antibody generated in rabbit against *E. coli* FtsZ^35^. Both rabbit-antibodies were subsequently detected with the secondary Peroxidase AffiniPure Donkey Anti-Rabbit IgG (H + L) (Jackson ImmunoResearch). mNG was detected with anti-mNG (Chromotek) and secondary goat anti-mouse IgG (H + L) HRP (Invitrogen). The conjugated horse radish peroxidase (HRP) of the secondary antibody was subsequently visualised with the Pierce™ ECL Western blotting substrate (Thermo Scientific) in a Licor Odyssey machine (700 nm channel for the Precision Plus Protein Dual Color Standards ladder (Bio-Rad), chemiluminescence channel for HRP activity).

### qPCR

The amount of mNG encoding mRNAs was established with qPCR. Total RNA was extracted from exponentially growing cells expressing mNG-v2-sfTq2^ox^, mNG-v5-sfTq2^ox^ and mNG-v6-sfTq2^ox^. Cells were harvested by centrifugation, lysed by cryogenic pulverisation and RNA was isolated from the lysate by phenol–chloroform isoamyl alcohol extraction, followed by isopropanol precipitation^37,38^. The concentration of extracted RNA was determined with a Nanodrop machine, and equal amounts were used as the template for cDNA synthesis with the First Strand cDNA Synthesis Kit (Thermo Scientific), implementing the random hexamer primers provided in the kit for amplification. The cDNA functioned subsequently as a template for a qPCR, with primers amplifying part of the mng sequence. For normalisation, established primers amplifying *idnT* were used^22^ in the DyNAmo HS SYBR Green qPCR Kit (Thermo Scientific). Three technical replicates per cDNA reaction and three technical replicates per qPCR resulted in nine qPCR reactions per sample. Normalisation was done by subtracting the mean threshold cycle (Ct) of the three replicate qPCRs per cDNA for *idnT* from the mean Ct for *mng* of the same cDNA template (ΔCt method). Values are negative as the threshold cycles for *mng* were reached earlier than those for *idnT.*

### Predicting translation efficiencies and mRNA structures in silico

Translation efficiency calculations were performed with RBS Calculator (version 2.1) accessed through https://salislab.net/software/predict_rbs_calculator, with standard settings and host organism *Escherichia coli* str. K-12 substr. MG1655^18^. Inserted sequences used for calculations span from the start of transcription until the fiftieth codon (in the T50 for sfTq2^ox^ variants and D50 for mNG variants). The exact input sequences can be found following Supplementary Table S1 in the supplementary materials.

### FRET experiment

FRET experiments were performed as previously described^11,39^. In short: LMC500 cells transformed with two plasmids (Supplementary Table S2) were grown to a steady state in GB1 medium supplied with ampicillin and chloramphenicol (for plasmid maintenance) at 28 °C. Subsequently, the cultures were diluted to an OD_450nm_ of 0.05 and induced with 15 µM IPTG (50 µM IPTG for sfTq2^oxopt^ and mNG references) for two mass doublings and then fixed with 2.8% formaldehyde and 0.04% glutardialdehyde (final concentrations). Fixed cells were washed with PBS and kept at RT for overnight maturation of the fluorescent proteins. Emission spectra were measured with the Spectrofluorometer Quantamaster 2000-4 (Photon Technology International) and unmixed as previously published^39^. For the purpose of this experiment, we used a known pair of interacting proteins: RodA and PBP2^23^. RodA with GlpT was the negative control, since it was previously proven that those two proteins do not interact^23^.

## Supplementary Information

Supplementary Information.

## Data Availability

The datasets generated and/or analysed during the current study are available from the corresponding author on reasonable request.
