# Supplementary material for: Optimising expression of the large dynamic range FRET pair mNeonGreen and superfolder mTurquoise2ox for use in the Escherichia coli cytoplasm
Source: Sci Rep. 2022 Oct 26;12:17977. doi: 10.1038/s41598-022-22918-2 (PMC9606377; doi:10.1038/s41598-022-22918-2)
Supplement: Supplementary file 1 — Supplementary Information. [file 41598_2022_22918_MOESM1_ESM.pdf]

Optimising expression of the large dynamic range FRET pair mNeonGreen and superfolder mTurquoise2^{ox} for use in the *Escherichia coli* cytoplasm.

Authors: Laureen M.Y. Mertens and Tanneke den Blaauwen*

Bacterial Cell Biology and Physiology, Swammerdam Institute for Life Science, University of Amsterdam, Science Park 904, 1098 XH Amsterdam, The Netherlands
* Correspondence: t.denblaauwen@uva.nl; Tel.: +31-20-525-3852

Supplementary Data

Figure S1: Standard operating procedure for fluorescent protein expression improvements in bacteria.

Table S1: Translation initiation rates as predicted by RBS Calculator (SalisLab) for Methionine1 and Methionine10 of different mNG and sfTq2^{ox} versions and RBSs

Figure S2: Original western blots

Figure S3: Representative microscopy images of mNeonGreen-v1-v6

Figure S4: Localisation of mNG-FtsZ fusions and mNG western blots

Figure S5: Representative microscopy images of all ribosome binding site related mNG versions in overnight matured cells

Table S2: Description of used FRET samples

Table S3: Summary of calculated FRET efficiencies (Ef_A) from spectral FRET with the FRET-pair RBS^{short} mNG-v4 and sfTq2^{oxopt}

Figure S6: Representative microscopy images of FRET samples

Figure S7: Unmixing of FRET experiments

Figure S8: mNeonGreen maturation upon fixation

Table S4: List of plasmids used in this study and the cloning procedures.

Table S5: List of used primers

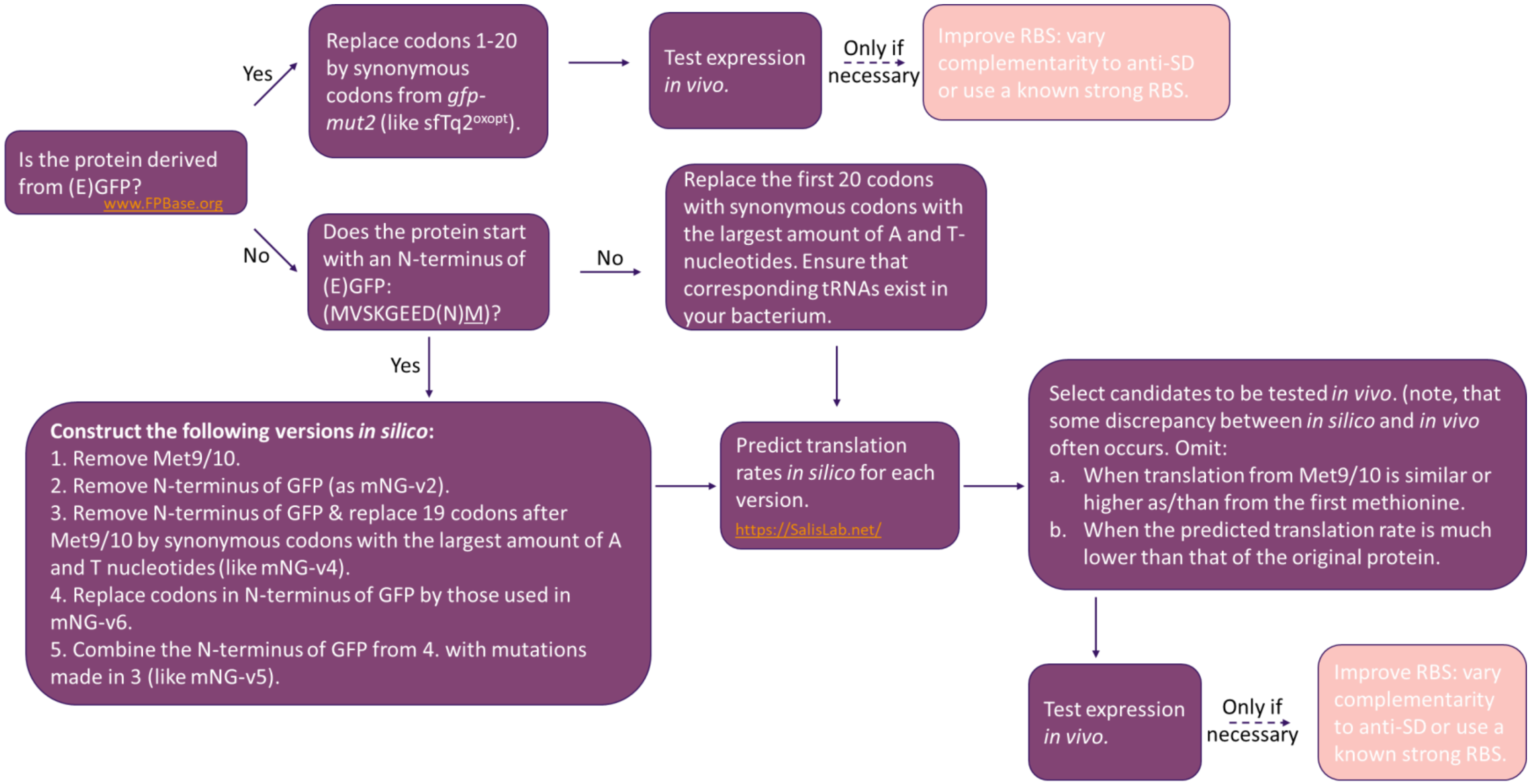

Figure S1: Standard operating procedure for fluorescent protein expression improvements in bacteria. This flowchart can be used as a guideline when optimizing the expression of fluorescent proteins in a bacterial species. Some remarks: Bacteria is written with an asterisk as we suspect the recommendations will work for many, but not all bacterial species: we recommend researchers to examine the specific characteristics of the translation machinery of their organism of choice. Sequences of sfTq2^{oxopt} and mNG-versions referred to in the flow chart can be found in Fig. 1 and Fig. 2 of the main manuscript. www.FPBase.org is an excellent resource for researchers interested in fluorescent proteins. For translation rate predictions, use the RBS calculator accessible through: https://salislab.net/software/predict_rbs_calculator[1]. Met9/10: This is Met10 in mNG; in some fluorescent proteins Asn9 is omitted, causing Met10 to be Met9.

Table S1: Translation initiation rates as predicted by RBS Calculator (SalisLab)[a] for Methionine1 (Met1) and Methionine10 (Met10) of different mNG and sfTq2^{ox} versions and RBSs[1].

	Start codon	Translation Initiation Rate (au)	ΔG_{total} (kcal/mol)	$\Delta G_{mRNA-rRNA}$ (kcal/mol)	$\Delta G_{spacing}$ (kcal/mol)	$\Delta G_{stacking}$ (kcal/mol)	$\Delta G_{standby}$ (kcal/mol)	ΔG_{start} (kcal/mol)	ΔG_{mRNA} (kcal/mol)
**mNG-v1**	Met1	440.54	2.29	-16.26	0	0	7.11	-2.76	-14.08
	Met10	147.38	4.72	-20.01	0.01	0	0.14	-2.76	-27.65
**mNG-v2**	~~Met1[b]~~	-	-	-	-	-	-	-	-
	Met10	372.97	2.66	-10.19	0	0	4.75	-2.76	-10.74
**mNG-v3**	Met1	381.46	2.61	-16.26	0	0	7.11	-2.76	-14.4
	Met10	89.6	5.83	-14.44	0.01	0	0	-2.76	-23.14
**mNG-v4**	~~Met1~~	-	-	-	-	-	-	-	-
	Met10	399.01	2.51	-12.55	0	0	7.11	-2.76	-10.59
**mNG-v5**	Met1	421.16	2.39	-12.55	0	0	7.11	-2.76	-10.47
	Met10	7576.18	-4.03	-18.22	1.73	0	1.27	-2.76	-14.56
**mNG-v6**	Met1	159.32	4.55	-12.6	0	0	7.11	-2.76	-12.68
	Met10	313.08	3.05	-18.22	1.73	0	1.27	-2.76	-21.64
**mNG-v5 M1K**	~~Met1~~	-	-	-	-	-	-	-	-
	Met10	13060.13	-5.24	-18.22	1.73	0	1.27	-2.76	-13.35
**mNG-v5 M10I**	Met1	421.16	2.39	-12.55	0	0	7.11	-2.76	-10.47
	~~Met10[b]~~	-	-	-	-	-	-	-	-
**RBS^{short}-mNG-v4**	~~Met1~~	-	-	-	-	-	-	-	-
	Met10	669.51	1.36	-4.85	0	0	2.06	-2.76	-6.79
**RBS^{long}-mNG-v4**	~~Met1~~	-	-	-	-	-	-	-	-
	Met10	4878.3	-3.06	-8.14	1.73	0	0.14	-2.76	-6.59
	Met1	23.76	8.78	-3.41	2.4	0	0	-2.76	-13.26

**BS-5'UTR-ymNG$_{WT}$**[c][2]	Met10	5.79	11.91	-12.63	1.52	0	4.41	-2.76	-21.36
**BS-5'UTR-ymNG$_{RM}$**[c][2]	Met1	21.82	8.97	-1.72	2.4	0	0	-2.76	-11.76
	Met10	1650.04	-0.65	-14.65	1.73	0	0.02	-2.76	-15.63
**sfTq2^{ox}**	Met1	1.48	14.95	-12.55	0.00	0.12	7.11	-2.76	-23.03
**sfTq2^{oxopt}**	Met1	267.32	3.40	-13.75	0.00	0.12	7.11	-2.76	-12.68

[a] Calculations were performed with RBS Calculator version 2.1 accessed through https://salislab.net/software/predict_rbs_calculator. Inserted sequences used for calculations can be found below the table.

[b] Strikethrough (~~Met1~~ or ~~Met10~~) means that this methionine is not present in the mNG-variant.

[c] BS-5'UTR means 5' Untranslated region from pBluescript II SK + of transcripts from lac promotor. ymNG$_{WT}$: mNG with its codons optimized according to codon usage in *Saccharomyces cerevisiae.* [2] ymNG$_{RM}$: RNA modified ymNG$_{WT}$[2].

Input sequences in RBS Calculator for each of the constructs:

5'-UTR is in lowercase. Start codons are coloured: M1 of mNG: dark green, M10 of mNG: light green and M1 of sfTq2$^{ox(opt)}$ in blue. Mutations M1K and M10I have been underlined. Input sequence spans from codon adjacent to -10' of lac promoter until the codon of K50. The 5'-UTR of the two ymNG variants is the 5'-UTR between the lac-promoter and *lacZ* pBluescript II SK (Stratagene), as described in the paper in which they were established[2].

mNG-v1
tgtggaattgtgagcggataacaatttcacacaggaaacagacc ATG GTG AGC AAG GGC GAG GAG GAT AAT ATG GCC TCT CTC CCA GCG ACA CAT GAG TTA CAC ATC TTT GGC TCC ATC AAC GGT GTG GAC TTT GAC ATG GTG GGT CAG GGC ACC GGC AAT CCA AAT GAT GGT TAT GAG GAG TTA AAC CTG AAG

mNG-v2
tgtggaattgtgagcggataacaatttcacacaggaaacagacc ATG GCC TCT CTC CCA GCG ACA CAT GAG TTA CAC ATC TTT GGC TCC ATC AAC GGT GTG GAC TTT GAC ATG GTG GGT CAG GGC ACC GGC AAT CCA AAT GAT GGT TAT GAG GAG TTA AAC CTG AAG

mNG-v3
tgtggaattgtgagcggataacaatttcacacaggaaacagacc ATG GTA AGT AAA GGT GAA GAG GAT AAC ATG GCC TCT CTC CCA GCG ACA CAT GAG TTA CAC ATC TTT GGC TCC ATC AAC GGT GTG GAC TTT GAC ATG GTG GGT CAG GGC ACC GGC AAT CCA AAT GAT GGT TAT GAG GAG TTA AAC CTG AAG

mNG-v4

tgtggaattgtgagcggataacaatttcacacaggaaacagacc ATG GCA TCA TTA CCA GCA ACA CAT GAA TTA CAT ATT TTT GGT TCT ATT AAT GGT GTT GAT TTT GAC ATG GTG GGT CAG GGC ACC GGC AAT CCA AAT GAT GGT TAT GAG GAG TTA AAC CTG AAG

mNG-v5

tgtggaattgtgagcggataacaatttcacacaggaaacagacc ATG GTT AGT AAA GGA GAA GAA GAT AAT ATG GCA TCA TTA CCA GCA ACA CAT GAA TTA CAT ATT TTT GGT TCT ATT AAT GGT GTT GAT TTT GAC ATG GTG GGT CAG GGC ACC GGC AAT CCA AAT GAT GGT TAT GAG GAG TTA AAC CTG AAG

mNG-v6

tgtggaattgtgagcggataacaatttcacacaggaaacagacc ATG GTT AGT AAA GGA GAA GAA GAT AAT ATG GCC TCT CTC CCA GCG ACA CAT GAG TTA CAC ATC TTT GGC TCC ATC AAC GGT GTG GAC TTT GAC ATG GTG GGT CAG GGC ACC GGC AAT CCA AAT GAT GGT TAT GAG GAG TTA AAC CTG AAG

mNG-v5 M1K

tgtggaattgtgagcggataacaatttcacacaggaaacagacc AAG GTT AGT AAA GGA GAA GAA GAT AAT ATG GCA TCA TTA CCA GCA ACA CAT GAA TTA CAT ATT TTT GGT TCT ATT AAT GGT GTT GAT TTT GAC ATG GTG GGT CAG GGC ACC GGC AAT CCA AAT GAT GGT TAT GAG GAG TTA AAC CTG AAG

mNG-v5 M10I

tgtggaattgtgagcggataacaatttcacacaggaaacagacc ATG GTT AGT AAA GGA GAA GAA GAT AAT ATA GCA TCA TTA CCA GCA ACA CAT GAA TTA CAT ATT TTT GGT TCT ATT AAT GGT GTT GAT TTT GAC ATG GTG GGT CAG GGC ACC GGC AAT CCA AAT GAT GGT TAT GAG GAG TTA AAC CTG AAG

RBS^{long}-mNG-v4

tgtggaattgtgagcggataacaagttagtaaaggagaagaagataat ATG GCA TCA TTA CCA GCA ACA CAT GAA TTA CAT ATT TTT GGT TCT ATT AAT GGT GTT GAT TTT GAC ATG GTG GGT CAG GGC ACC GGC AAT CCA AAT GAT GGT TAT GAG GAG TTA AAC CTG AAG

RBS^{short}-mNG-v4

tgtggaattgtgagcggataacaatttggagaagaagatagacc ATG GCA TCA TTA CCA GCA ACA CAT GAA TTA CAT ATT TTT GGT TCT ATT AAT GGT GTT GAT TTT GAC ATG GTG GGT CAG GGC ACC GGC AAT CCA AAT GAT GGT TAT GAG GAG TTA AAC CTG AAG

BS-5'UTR-$ymNG_{WT}$

acaccttaacactcgcctattgttaaagtgtgtcctttgtcga ATG GTC AGT AAG GGT GAA GAA GAT AAC ATG GCT TCT TTG CCA GCT ACT CAT GAA TTG CAT ATT TTC GGT TCC ATC AAC GGT GTT GAT TTC GAT ATG GTT GGT CAA GGT ACT GGT AAT CCA AAT GAT GGT TAC GAA GAA TTG AAC TTG AAG

BS-5'UTR-ymNG$_{RM}$

acaccttaacactcgcctattgttaaagtgtgtcctttgtcga ATG GTT TCA AAG GGA GAA GAA GAT AAT ATG GCA TCA TTA CCA GCA ACA CAT GAA TTG CAT ATT TTC GGT TCC ATC AAC GGT GTT GAT TTC GAT ATG GTT GGT CAA GGT ACT GGT AAT CCA AAT GAT GGT TAC GAA GAA TTG AAC TTG AAG

sfTq2^{ox}

tgtggaattgtgagcggataacaatttcacacaggaaacagacc ATG GTG AGC AAG GGC GAG GAG CTG TTC ACC GGG GTG GTG CCC ATC CTG GTC GAG CTG GAC GGC GAC GTA AAC GGC CAC AAG TTC AGC GTG CGC GGC GAG GGC GAG GGC GAT GCC ACC AAT GGC AAG CTG ACC CTG AAG TTC ATC TGC ACC ACC

sfTq2^{oxopt}

tgtggaattgtgagcggataacaatttcacacaggaaacagacc ATG GTT AGT AAA GGA GAA GAA CTT TTC ACT GGA GTT GTC CCA ATT CTT GTT GAA TTA GAT GGC GAC GTA AAC GGC CAC AAG TTC AGC GTG CGC GGC GAG GGC GAG GGC GAT GCC ACC AAT GGC AAG CTG ACC CTG AAG TTC ATC TGC ACC ACC

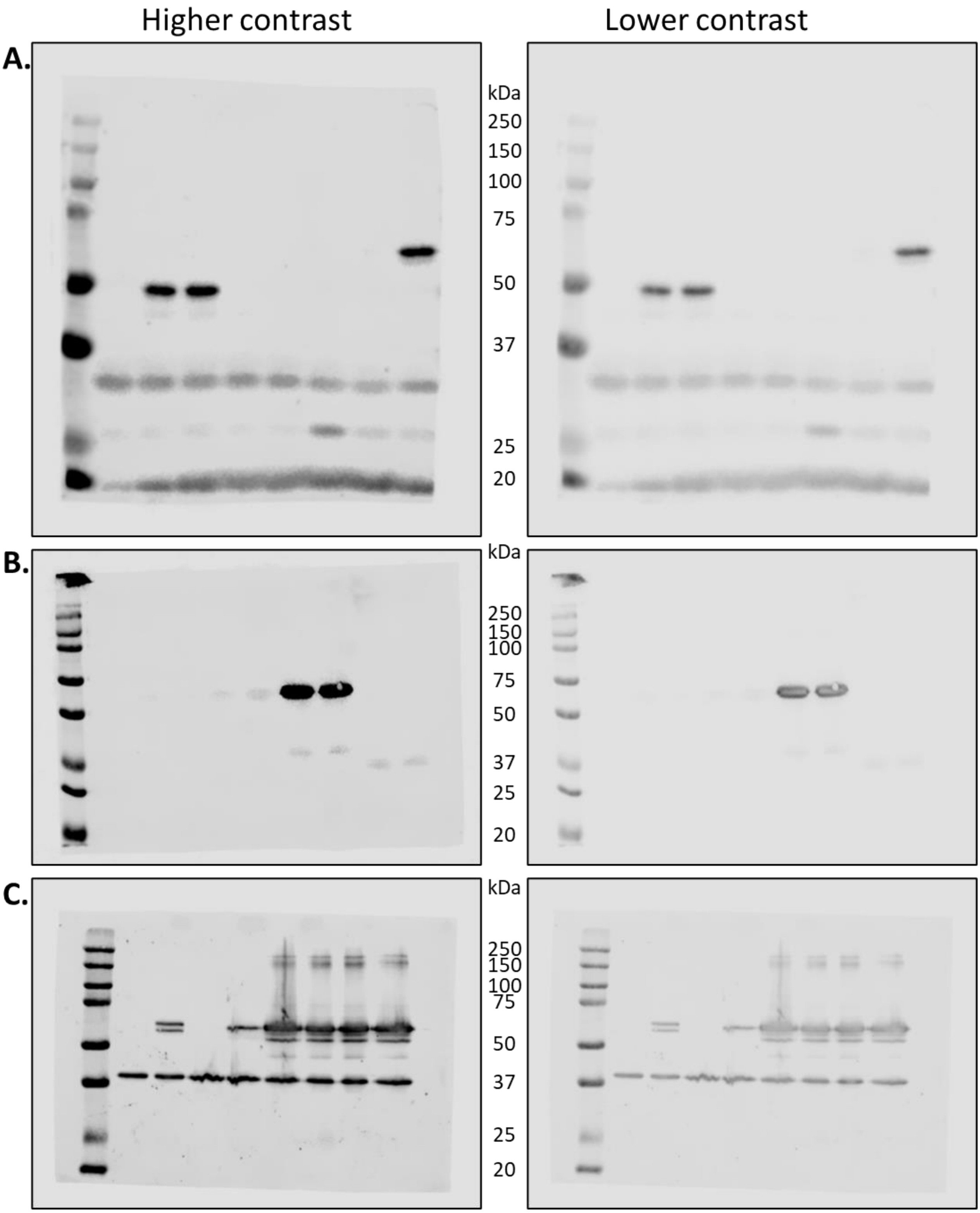

Figure S2: Original western blots. For each blot a higher and lower contrast image are shown side-by-side. Mass of each ladder bands is indicated in the middle. **(A)** original blot from main text Fig. 1D, **(B)** Original blot from main text Fig 2D, **(C)** Original blot from Supplementary Fig. S4D.

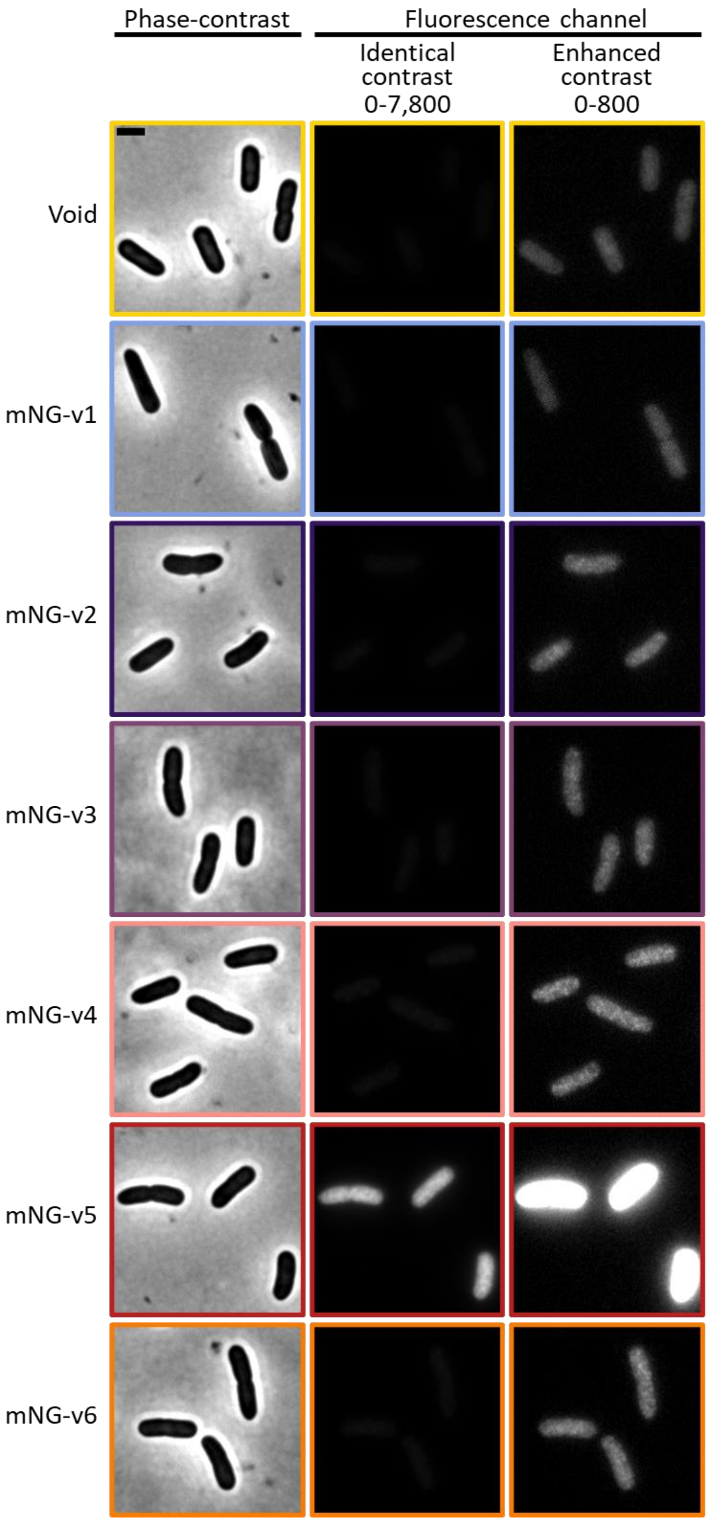

Figure S3: Representative microscopy images of mNeonGreen-v1-v6. Representative microscopy images of all 6 version of mNG in overnight matured cells (these samples are also shown as fluorescence per μm^3 in main-text figure 2B). Phase contrast images are shown on the left with the corresponding fluorescence images on the right. The numbers above the fluorescence images are the brightness and contrast settings for the image. Scale bar equals 2 µm.

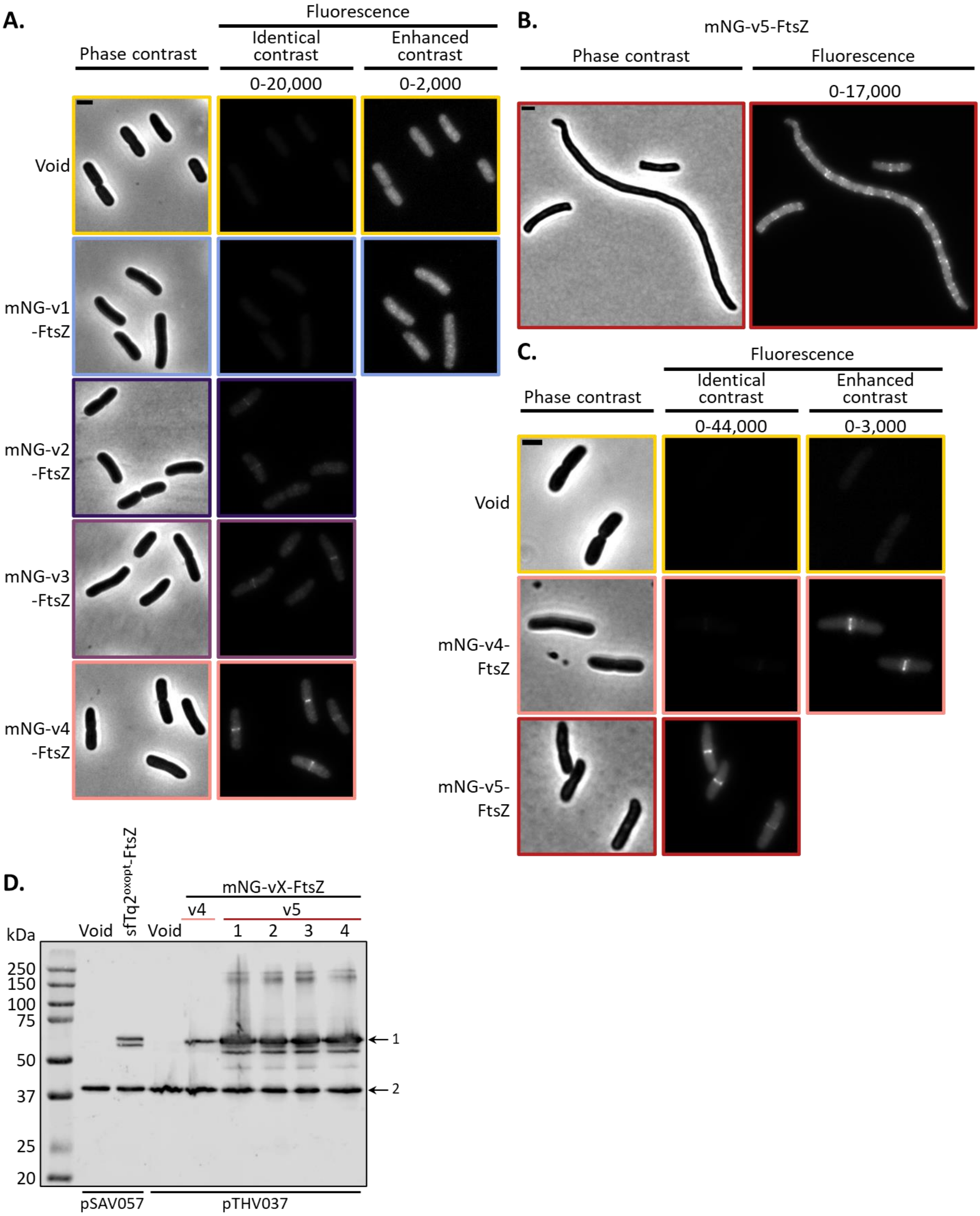

Figure S4: Localisation of mNG-FtsZ fusions and mNG western blots. (A), (B) and (C) Three separate experiments expressing different mNG-FtsZ fusions. Samples in (A) and (B) were induced with 15 µM IPTG for two mass doublings. mNG-v5-FtsZ causes severe defects to cells (filamentation and contorted poles). The experiment was therefore repeated without IPTG induction (see C). Scale bars equal 2 µm. **(D)** Western blot labelled with anti-FtsZ, allows to determine ratio of amounts of sfTq2^{oxopt}-FtsZ and mNG-FtsZ produced. sfTq2^{oxopt}-FtsZ is shown next to its corresponding empty vector (pSAV057), whereas the mNG-FtsZ fusions are shown next to their corresponding void plasmid pTHV037. Four replicates of mNG-v5-FtsZ were loaded. Arrow 1 indicates the height expected for a FP-FtsZ fusion, arrow 2 indicates the height expected for unfused FtsZ (expressed from the

chromosome). The material represented by the double band shown for sfTq2^{oxopt}-FtsZ is more than the band of mNG-v4-FtsZ – confirming that the ratio is towards sfTq2^{oxopt}, whereas the bands of mNG-v5-FtsZ are clearly too much for a good ratio with sfTq2^{oxopt}-FtsZ. In addition to this, mNG-v5-FtsZ show a lot of background signal, the smaller bands are possibly degradation products, but also bands higher than indicated by arrow 1. Original western blot shown in Supplementary Fig. S2C.

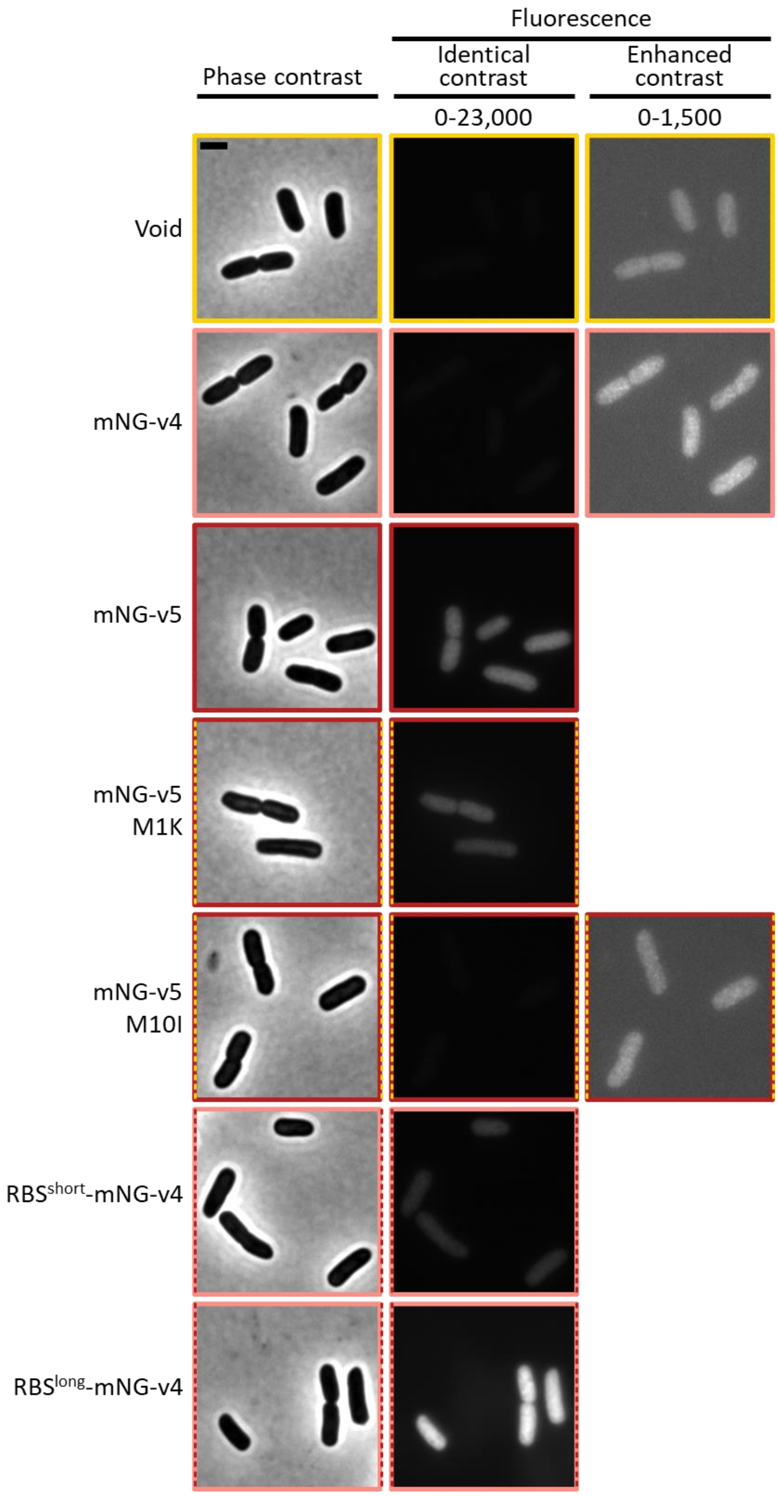

Figure S5: Representative microscopy images of all ribosome binding site related mNG versions in overnight matured cells (these samples are also shown as fluorescence per µm^3 in main-text figure 3A). Phase contrast images are shown on the left with the corresponding fluorescence images on the right. The numbers above the fluorescence images are the brightness and contrast settings for the column of images below. Scale bar equals 2 µm.

Table S2: Description of used FRET samples

Sample name	Plasmid name and description of $P_{trcdown}$ controlled transcription unit	
	**Plasmid 1 (ampicillin^R)**	**Plasmid 2 (chloramphenicol^R)**
Blanco	pTHV037: void	pSAV057: void
RBS^{short}-mNG-v4 reference	pTEN018: RBS^{short}-mNG-v4	pSAV057: void
sfTq2^{oxopt} reference	pTHV037: void	pLMY005 sfTq2^{oxopt}
Tandem	pTHV037: void	pLMY038 RBS^{short}-mNG-v4–sfTq2^{oxopt}
Negative control	pLMY039: RBS^{short}-mNG-v4–RodA	pIDI012 sfTq2^{oxopt}–GlpT
RodA – PBP2	pLMY039: RBS^{short}-mNG-v4–RodA	pTEN002 sfTq2^{oxopt}–PBP2

Table S3: Summary of calculated FRET efficiencies (Ef_A) from spectral FRET with the FRET-pair RBS^{short} mNG-v4 and sfTq2^{oxopt}

Sample description		Proteins expressed		Ef_A (%)	SD (%)	N
		**Plasmid 1**	**Plasmid 2**			
Positive control	Tandem	Empty plasmid	mNG-v4-sfTq2^{ox}	64.4	2.1	4
Negative control	RodA-GlpT	mNG-v4-RodA	sfTq2^{oxopt}-GlpT	1.2	1.1	4
Biological interaction	RodA-PBP2	mNG-v4-RodA	sfTq2^{oxopt}-PBP2	20.2	1	4

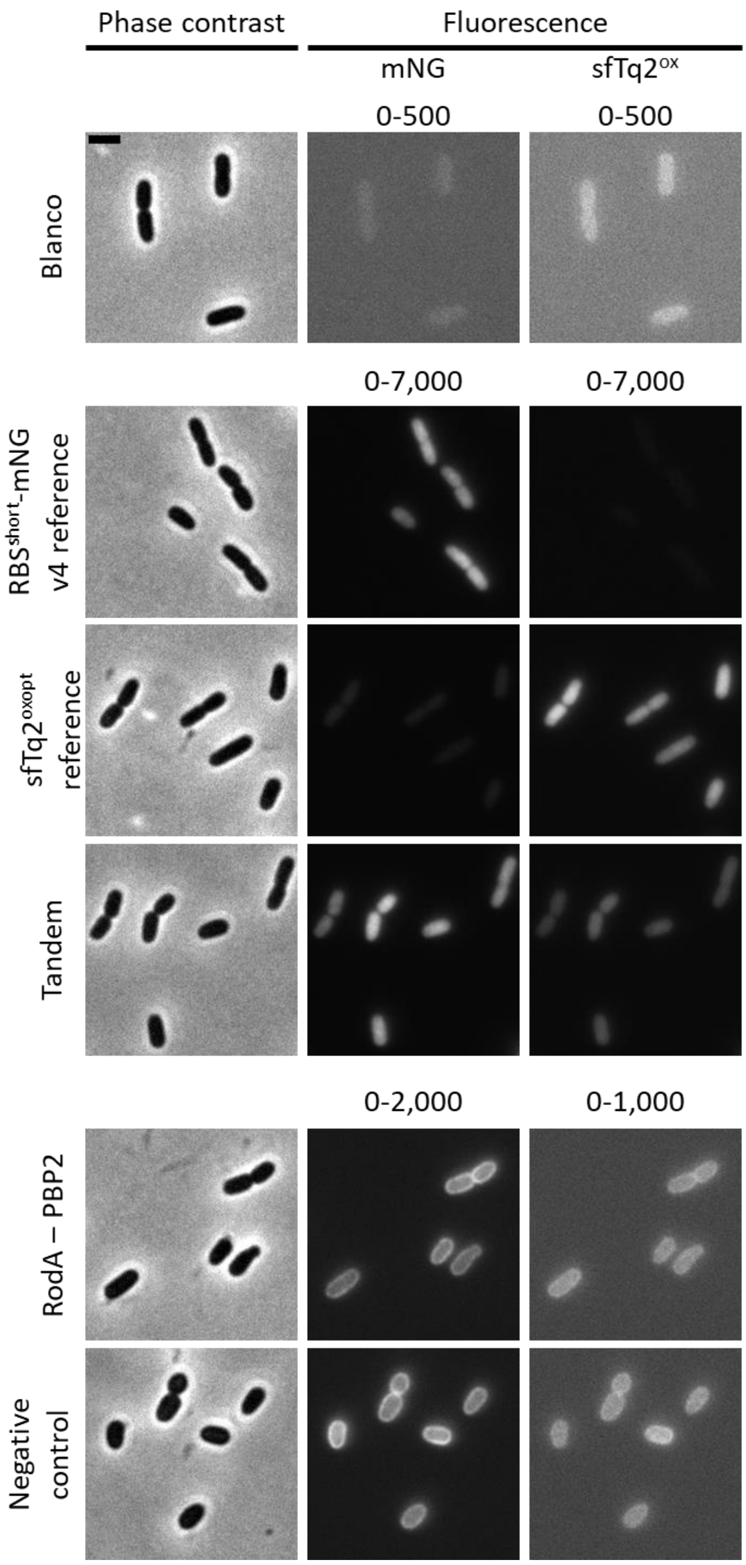

Figure S6 Representative microscopy images of FRET samples. Sample names correspond to table S2. The three images per sample are from left to right: phase contrast, corresponding fluorescence image from imaging with the GFP-B filter for mNG, and the fluorescence as imaged through the CFP filter for sfTq2. The numbers above the fluorescence images are the brightness and contrast settings for the images below that number. Scale bar equals 2 µm.

A.

Tandem #1

Ef_A = 67.2% ± 0.4%
Ef_D = 29.0 % ± 0.1 %
Ef_A/Ef_D = 2.31 ± 0.02

Tandem #2

Ef_A = 62.4% ± 0.4%
Ef_D = 29.3 % ± 0.1%
Ef_A/Ef_D = 2.12 ± 0.02

Tandem #3

Ef_A = 64.4% ± 0.3%
Ef_D = 31.5% ± 0.1 %
Ef_A/Ef_D = 2.04 ± 0.01

Tandem #4

Ef_A = 63.3% ± 0.3%
Ef_D = 31.6 % ± 0.1 %
Ef_A/Ef_D = 2.01 ± 0.01

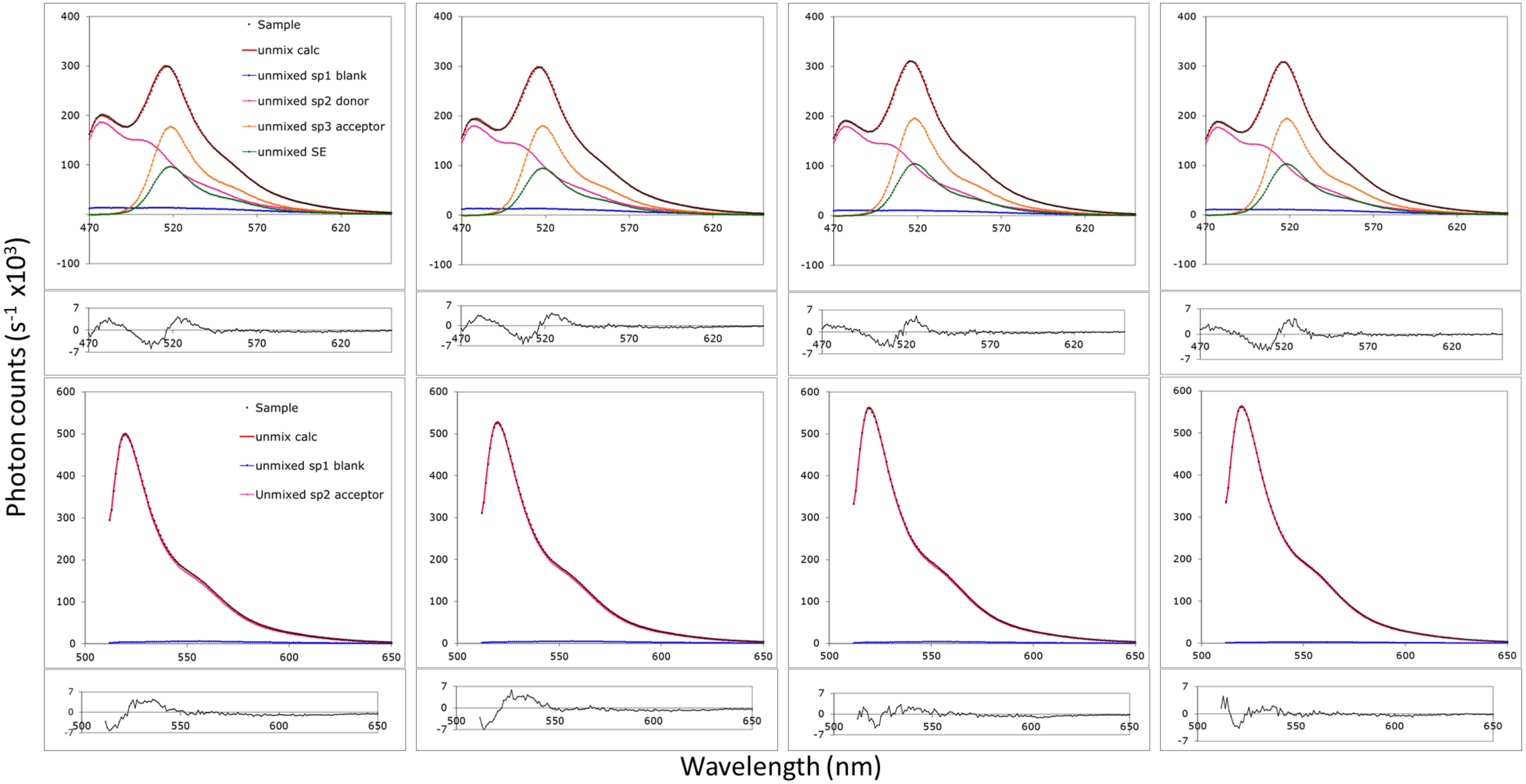

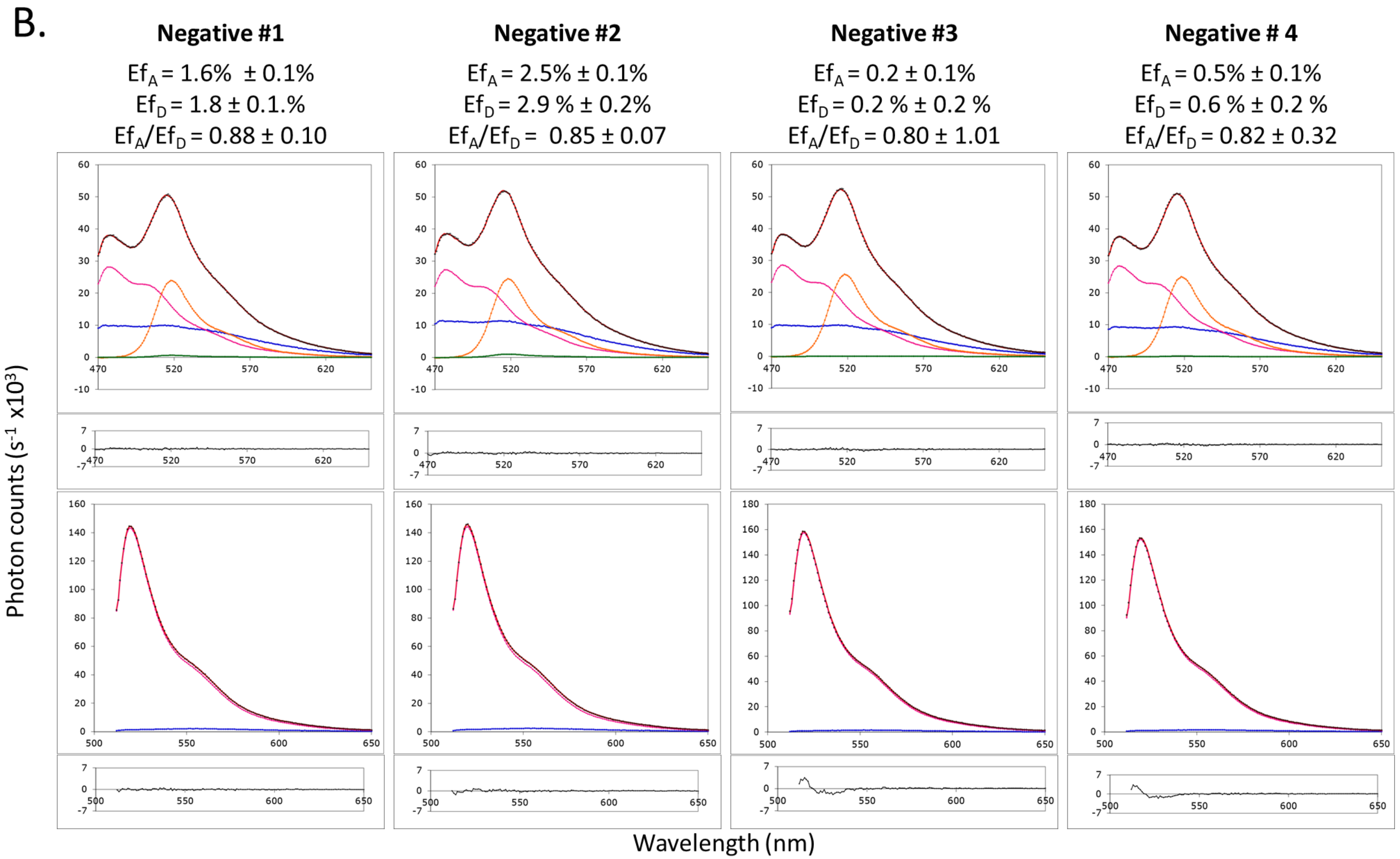
B.
Negative #1
EfA = 1.6% ± 0.1%
EfD = 1.8 ± 0.1.%
EfA/EfD = 0.88 ± 0.10
Negative #2
EfA = 2.5% ± 0.1%
EfD = 2.9 % ± 0.2%
EfA/EfD = 0.85 ± 0.07
Negative #3
EfA = 0.2 ± 0.1%
EfD = 0.2 % ± 0.2 %
EfA/EfD = 0.80 ± 1.01
Negative # 4
EfA = 0.5% ± 0.1%
EfD = 0.6 % ± 0.2 %
EfA/EfD = 0.82 ± 0.32
Photon counts (s-1 x103)
Wavelength (nm)

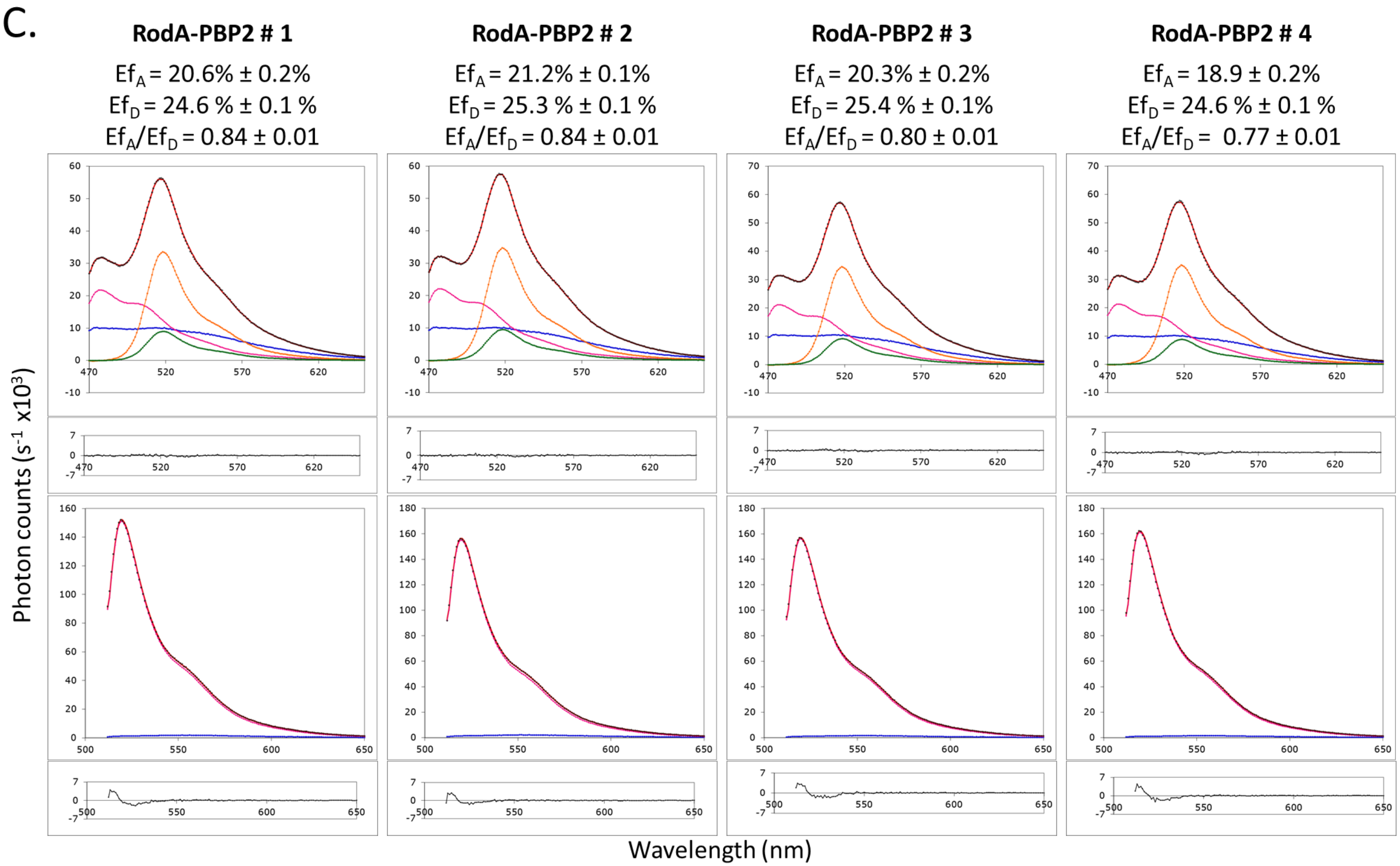
C.
RodA-PBP2 # 1
Ef_A = 20.6% ± 0.2%
Ef_D = 24.6 % ± 0.1 %
Ef_A/Ef_D = 0.84 ± 0.01
RodA-PBP2 # 2
Ef_A = 21.2% ± 0.1%
Ef_D = 25.3 % ± 0.1 %
Ef_A/Ef_D = 0.84 ± 0.01
RodA-PBP2 # 3
Ef_A = 20.3% ± 0.2%
Ef_D = 25.4 % ± 0.1%
Ef_A/Ef_D = 0.80 ± 0.01
RodA-PBP2 # 4
Ef_A = 18.9 ± 0.2%
Ef_D = 24.6 % ± 0.1 %
Ef_A/Ef_D = 0.77 ± 0.01
Photon counts (s-1 x103)
Wavelength (nm)

Figure S7: Unmixing plots of individual FRET experiments. Individual unmixing plots of each individual sample – four replicates each- of **(A)** Tandems, **(B)** Negative control and **(C)** RodA-PBP2. Sample names correspond to table S2. The unmixing plots of Tandem #1 show the legend for all lines. Above the plots of each sample, calculated acceptor fret (Ef_A), donor FRET (Ef_D) with their standard deviations and the Ef_A/Ef_D ratio are shown.

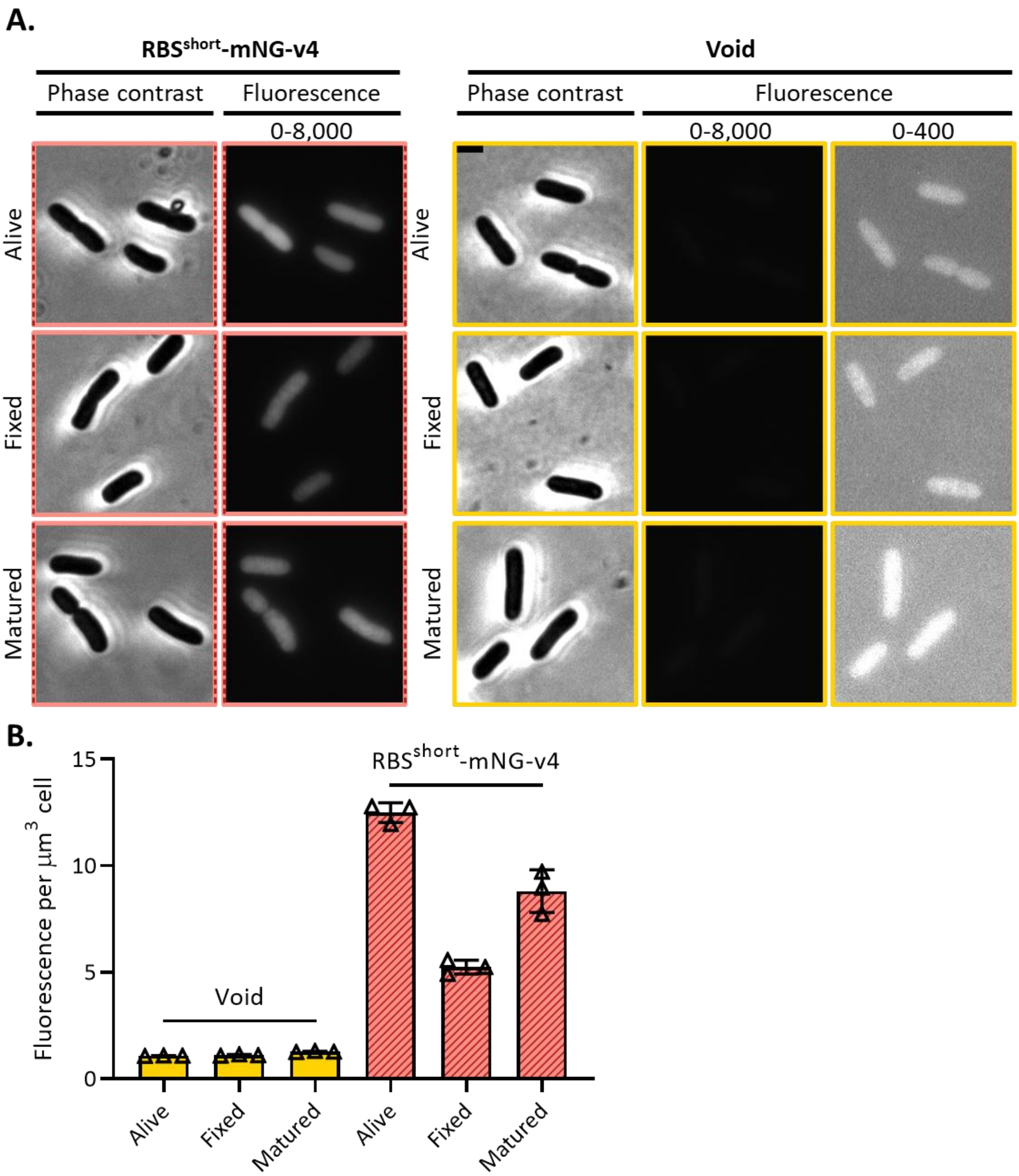

Figure S8: mNeonGreen maturation upon fixation. Fluorescence of mNeonGreen decreases upon fixation with FAGA. For RBS^{short}-mNG-v4 fluorescence per µm^3 drops to aproximately 40% of the fluorescence of living cells imaged shortly after fixation, and after overnight incubation at room temperature it increases to ~70% of the fluorescence per µm^3 cell volume in living cells. **(A)** Representative microscopy images of samples, **(B)** Fluorescence per µm^3 cell for cells expressing no mNG and cells expressing RBS^{short}-mNG-v4 in living cells (alive), shortly after fixation (fixed) and overnight incubated fixed cells (matured).

Table S3: List of plasmids used in this study and cloning procedures.

Plasmid	Description						Reference
pSAV057	Expression vector, derived from pTRC99A, with a weakened Ptrcdown promotor, pBR322 origin, ampicillin resistance (*bla*).						[3]
pTHV037	Expression vector, derived from pTRC99A, with a weakened Ptrcdown promotor, p15 origin, chloramphenicol resistance (*cat)*.						[4]
	**Expressed protein**	**Backbone**	**Cloning method***	**Restriction enzymes**	**Paternal plasmid(s)**	**Primers**	
pNM001	mNG-v2	pSAV057					[5]
pNM042	sfTq2^{ox}	pSAV057					[6]
pNM045	mNG-v2-sfTq2^{ox}	pSAV057					[6]
pNM051	sfTq2^{ox}-EFSRDL-FtsZ	pSAV057					[6]
pNM088	OmpA-(SA-1)-177-LEDPPAEL-sfTq2^{ox}	pTHV037					[6]
pWA004	mKO-PBP2	pSAV057					[7]
pXL29	mKO-(GGS)$_2$-GlpT	pSAV057					[8]
pXL30	mCh-(GGS)$_2$-GlpT	pTHV037					Gift from Xiaolong Liu
pXL40	mCh-RodA^{Q207R}	pTHV037					[8]
pUA66-PrprA-mNG		pUA66					[9]
**sfTq2^{oxopt}-constructs**							
pLMY004	sfTq2^{oxopt}	pSAV057	SDM		pNM042	LM035 + LM036	This study
pLMY005	sfTq2^{oxopt}-EFSRDL-FtsZ	pSAV057	SDM		pNM051	LM035 + LM036	This study
pLMY043	OmpA-(SA-1)-177-LEDPPAEL-sfTq2^{oxopt}	pTHV037	PCR, R+L	BamHI + BsrGI	pNM088<br>pLMY005	 LM081 + LM082	This study
pTEN002	sfTq2^{oxopt}-PBP2	pSAV057	R+L	EcoRI + SphI	pWA004<br>pLMY005		This study

**mNG-FtsZ constructs**							
pNM200	mNG-v1-EFSRDL-FtsZ	pTHV037	PCR, R+L	BsrGI + NcoI	pSAV078<br>pNM002	 NM086 + NM234	This study
pNM201	mNG-v2-EFSRDL-FtsZ	pTHV037	PCR, R+L	NcoI + XbaI	pSAV073<br>pNM001	 NM253 + NM255	This study
pLMY037	mNG-v3-EFSRDL-FtsZ	pTHV037	PCR, R+L	BsrGI + NcoI	pNM201<br>pUA66-PrprA-mNG	-<br>LM085 + LM054	This study
pLMY007	mNG-v4-EFSRDL-FtsZ	pTHV037	SDM		pNM201	LM088A + LM089A	This study
pLMY033	mNG-v5-EFSRDL-FtsZ	pTHV037	SDM		pLMY007	LM110 + LM111	This study
**Tandem constructs**							
pLMY006	mNG-v4-sfTq2^{ox}	pSAV057	SDM		pNM045	LM088A + LM089A	This study
pLMY032	mNG-v5-sfTq2^{ox}	pSAV057	SDM		pLMY006	LM110 + LM111	This study
pTEN005	mNG-v6-sfTq2^{ox}	pSAV057	SDM		pNM045	LM111 + LM123	This study
pTEN022	mNG-v5^{M10I}-sfTq2^{ox}	pSAV057	SDM		pLMY032	LM124 + LM125	This study
pLMY038	RBS^{short}-mNG-v4-sfTq2^{ox}	pSAV057	SDM		pLMY006	LM137 + LM138	This study
**GlpT constructs**							
pIDI012	sfTq2^{oxopt}-GlpT	pSAV057	GA		pXL029<br>pLMY005	JV47+JV48<br>JV49+JV50	This study
**RodA constructs**							
pIDI005	mNG-v4-RodA^{Q207R}	pTHV037	GA		pXL40	JV30 + JV31	This study
pIDI004	mNG-v4-RodA	pTHV037	SDM		pLMY006<br>pIDI005	JV28 + JV29<br>JV39 + JV40	This study
pTEN005	mNG-v5-RodA	pTHV037	SDM		pIDI004	LM110 + LM111	This study
pLMY039	RBS^{short}mNG-v4-RodA	pTHV037	SDM		pIDI004	LM137 + LM138	This study
**Unfused mNG constructs**							
pTEN014	mNG-v1	pTHV037	PCR, R+L	HindIII + NcoI	pIDI011		This study

pTEN015	mNG-v2	pTHV037	PCR, R+L	HindIII + NcoI	pNM200<br>pIDI011	LM102 + LM141	This study
pTEN016	mNG-v3	pTHV037	PCR, R+L	HindIII + NcoI	pNM201<br>pIDI011	LM078 + LM141	This study
pIDI011	mNG-v4	pTHV037	SDM, R+L	HindIII	pLMY037<br>pIDI005	LM085 + LM141<br>JV41 + JV42	This study
pTEN003	mNG-v5	pTHV037	SDM		pIDI011	LM110 + LM111	This study
pTEN004	mNG-v6	pTHV037	SDM		pTEN015	LM111 + LM123	This study
pTEN020	mNG-v5 M1K	pTHV037	SDM		pTEN003	TEN15 + TEN16	This study
pTEN021	mNG-v5 M10I	pTHV037	SDM		pTEN003	LM124 + LM125	This study
pTEN017	RBS^{long}mNG-v4	pTHV037	SDM		pIDI011	LM139 + LM140	This study
pTEN018	RBS^{short}mNG-v4	pTHV037	SDM		pIDI011	LM137 + LM138	This study

* **SDM**: site directed mutagenesis, R+L: restriction-ligation, **PCR, R+L**: PCR amplification of insert, followed by restriction-ligation, **GA**: Gibson assembly, **SDM, R+L**: site directed mutagenesis, followed by restriction-ligation to close plasmid prior to transformation.

Table S4: All used primers

Primer name	Sequence (5' -> 3')	Purpose
**Cloning primers**		
LM035	A**A**CT**T**TTCAC**T**GG**A**GT**T**GT**C**CC**A**AT**T**CT**T**GT**T**GA**AT**T**A**GA**T**GGCGACGTAAACGGCCAC	sfTq2^{ox} to sfTq2^{oxopt} FW
LM036	**T**GG**G**AC**A**AC**T**CC**A**GTGAA**A**AG**T**TC**T**TC**T**CC**T**TT**A**CT**A**ACCATGGTCTGTTTCCTGTGTG	sfTq2^{ox} to sfTq2^{oxopt} RV
LM054	TTGTACAGCTCGTCCATGCCCATC	mNG rv BsrGI
LM078	CAGACCATGGCCTCTCTCCCAG	mNG-v2 FW NcoI
LM081	AAAGGATCCCCCCGCTGAATTGATGGTTAGTAAAGGAGAAGAACTTTTCACTGG	FW-sfTq2^{oxopt} for ompA177 BamHI
LM082	AAACGAATTCCTTGTACAGCTCGTCCATGC	RV-sfTq2^{oxopt} for ompA177 EcoRI
LM085	ATACCATGGTAAGTAAAGGTGAAGAGGATAACATGGCC	mNG-v3 FW NcoI
LM088A	ACATGA**A**TTACA**T**AT**T**TTTGG**T**TC**T**AT**T**AA**T**GGTGT**T**GA**T**TTTGACATGGTGGGTCAGG	mNG-v2 to mNG-v4 FW
LM089A	CCAAA**A**AT**A**TGTAA**T**TCATGTGT**T**GCTGG**T**A**AT**GA**T**GCCATGGTCTGTTTCCTGTGTG	mNG-v2 to mNG-v4 RV
LM102	AAGTGATTGACGGCTACGGTGGATCCATGGTGAGCAAGGGCGAG	Amplify mNG-v1 NcoI
LM110	TAAAGGAGAAGAAGATAATATGGCATCATTACCAGCAACACATGAATTAC	mNG-v4 to mNG-v5 FW
LM111	CTTCTTCTCCTTTACTAACCATGGTCTGTTTCCTGTGTGAAATTGTTATC	mNG-v4 to mNG-v5 RV
LM137	TTT**GGAGAAGAAGAT**AGACCATGGCATCATTACCAGCAACAC	RBS^{short} FW upstream of mng-v4
LM138	TGGTCT**ATCTTCTTCTCC**AAATTGTTATCCGCTCACAATTCCACACATTATACGAG	RBS^{short} RV upstream of mng-v4
LM139	AA**GTTAGTAAAGGAGAAGAAGATAAT**ATGGCATCATTACCAGCAACACATGAATTAC	RBS^{long} FW upstream of mng-v4
LM140	**ATTATCTTCTTCTCCTTTACTAAC**TTGTTATCCGCTCACAATTCCACACATTATACGAG	RBS^{long} RV upstream of mng-v4
LM141	AAGGAAGCTTTTACTTGTACAGCTCGTCCATGCCCATC	mNG RV HindIII
JV28	CACACAGGAAACAGACCATGGCATCATTA	fragRodA-F
JV29	CCGTCATGTTGTTGTTCTTGTACAGCTCGTCCATGCCC	fragRodA-R

JV30	GCATGGACGAGCTGTACAAGAACAACAACATGAC	vectRodA-F
JV31	GTTGCTGGTAATGATGCCATGGTCTGTTTCCTGTGTGAAAT	vectRodA-R
JV39	GTTCTTCCTGATGCATGATTACCAGCGCCAGCGCGTAATGATGC	RodA wt mut Fw
JV40	GCATCATTACGCGCTGGCGCTGGTAATCATGCATCAGGAAGAAC	RodA wt mut Rv
JV41	GATCGAAGCTTCCTTGGCTGTTTTG	pIDI011 F
JV42	CGCCAAGCTTTTACTTGTACAGCTC	pIDI011 R
JV43	CTTTACCGATGTGATGGGCATGGACGAGCTGTAC	mNG GlpT VecFw
JV44	GTTGCTGGTAATGATGCCATGGTCTGTTTCCTGTG	mNG GlpT VecRv
JV45	CACAGGAAACAGACCATGGCATCATTACCAGCAAC	mNG GlpT FraFw
JV46	GTACAGCTCGTCCATGCCCATCACATCGGTAAAG	mNG GlpT FraRv
JV047	GACGAGCTGTACAAGGAATTCGGAGGATCTGG	mTq GlpT VecFw
JV048	GTTCTTCTCCTTTACTAACCATGGTCTGTTTCC	mTq GlpT VecRv
JV049	GGAAACAGACCATGGTTAGTAAAGGAGAAGAAC	mTq GlpT FraFw
JV050	CCAGATCCTCCGAATTCCTTGTACAGCTCGTC	mTq GlpT FraRv
TEN015	GGAAACAGACCAAGGTTAGTAAAGGAGAAGAAGATAATATGG	M1K fw
TEN016	CTAACCTTGGTCTGTTTCCTGTGTGAAATTGTTATCC	M1K rev
LM124	AAGATAATATAGCATCATTACCAGCAACACATG	M10I fw
LM125	ATGCTATATTATCTTCTTCTCCTTTACTAACCATGG	M10I rev
NM253	GGGGGACCATGGCCTCTCTCCCAGCGACACATGAGTTACAC	mNG-d9-NcoI
NM255	CAAACATGAGATCTCTAGAGAATTCCTTGTACAGCTCGTCCATGCC	XbaI-link-FtsZ-R
NM234	GGGGGACCATGGTGAGCAAGGGCGAG	Ncoi-*FP
NM86	GCGCGCAAGCTTTAGAATTCCTTGTACAGCT	NG-HindIII-R
**qPCR primers**		
Zhou-*ldnT*-F	CTGTTTAGCGAAGAGGAGATGC	qPCR *idnT*-F [10]
Zhou-*ldnT*-R	ACAAACGGCGGCGATAGC	qPCR *idnT*-R [10]
LM146	GCAGTTTGAAGATGGTGCCTCCC	qPCR *mng*-F
LM146A	GTCAGCGAGTTGGTCATCACAGG	qPCR *mng*-R

Bold: Nucleotide substitutions; Underlined: restriction site used for cloning purposes

References

1. Reis, A. C. & Salis, H. M. An Automated Model Test System for Systematic Development and Improvement of Gene Expression Models. *ACS Synth. Biol.* **9**, 3145–3156 (2020).
2. Hector, R. E., Mertens, J. A. & Nichols, N. N. Increased expression of the fluorescent reporter protein ymNeonGreen in *Saccharomyces cerevisiae* by reducing RNA secondary structure near the start codon. *Biotechnology Reports* **33**, e00697 (2022).
3. Den Blaauwen, T., Aarsman, M. E. G., Vischer, N. O. E. & Nanninga, N. Penicillin-binding protein PBP2 of *Escherichia coli* localizes preferentially in the lateral wall and at mid-cell in comparison with the old cell pole: Localization of PBP2. *Molecular Microbiology* **47**, 539–547 (2003).
4. Alexeeva, S., Gadella, T. W. J., Verheul, J., Verhoeven, G. S. & Den Blaauwen, T. Direct interactions of early and late assembling division proteins in Escherichia coli cells resolved by FRET: Bacterial division studied by spectral FRET. *Molecular Microbiology* **77**, 384–398 (2010).
5. Meiresonne, N. Y., van der Ploeg, R., Hink, M. A. & den Blaauwen, T. Activity-Related Conformational Changes in D,D -Carboxypeptidases Revealed by *In Vivo* Periplasmic Förster Resonance Energy Transfer Assay in *Escherichia coli*. *mBio* **8**, e01089-17 (2017).
6. Meiresonne, N. Y. *et al.* Superfolder mTurquoise2 ^{ox} optimized for the bacterial periplasm allows high efficiency *in vivo* FRET of cell division antibiotic targets. *Mol Microbiol* **111**, 1025–1038 (2019).
7. van der Ploeg, R. *et al.* Colocalization and interaction between elongasome and divisome during a preparative cell division phase in *Escherichia coli*: Preparing cell division. *Molecular Microbiology* **87**, 1074–1087 (2013).
8. Liu, X., Biboy, J., Consoli, E., Vollmer, W. & den Blaauwen, T. MreC and MreD balance the interaction between the elongasome proteins PBP2 and RodA. *PLoS Genet* **16**, e1009276 (2020).
9. Steenhuis, M. *et al.* Inhibition of autotransporter biogenesis by small molecules. *Mol Microbiol* **112**, 81–98 (2019).
10. Zhou, K. *et al.* Novel reference genes for quantifying transcriptional responses of Escherichia coli to protein overexpression by quantitative PCR. *BMC Molecular Biol* **12**, 18 (2011).
